# Time and age dependent regulation of neuroinflammation in a rat model of mesial temporal lobe epilepsy: Correlation with human data

**DOI:** 10.3389/fcell.2022.969364

**Published:** 2022-09-13

**Authors:** Sinem Erisken, George Nune, Hyokwon Chung, Joon Won Kang, Sookyong Koh

**Affiliations:** ^1^ Department of Pediatrics, Stanley Manne Children’s Research Institute, Ann & Robert H. Lurie Children’s Hospital of Chicago, Northwestern University School of Medicine, Chicago, IL, United States; ^2^ Department of Biomedical Engineering, McCormick School of Engineering, Northwestern University, Evanston, IL, United States; ^3^ Department of Neurology, University of Southern California, Los Angeles, CA, United States; ^4^ Department of Pediatrics, Children’s Hospital & Medical Center, University of Nebraska, Omaha, NE, United States; ^5^ Department of Pediatrics & Medical Science, Brain Research Institute, College of Medicine, Chungnam National University, Daejeon, South Korea

**Keywords:** epileptogenesis, inflammation, gene expression, microarray, status epilepticus, MAPK, glia

## Abstract

Acute brain insults trigger diverse cellular and signaling responses and often precipitate epilepsy. The cellular, molecular and signaling events relevant to the emergence of the epileptic brain, however, remain poorly understood. These multiplex structural and functional alterations tend also to be opposing - some homeostatic and reparative while others disruptive; some associated with growth and proliferation while others, with cell death. To differentiate pathological from protective consequences, we compared seizure-induced changes in gene expression hours and days following kainic acid (KA)-induced status epilepticus (SE) in postnatal day (P) 30 and P15 rats by capitalizing on age-dependent differential physiologic responses to KA-SE; only mature rats, not immature rats, have been shown to develop spontaneous recurrent seizures after KA-SE. To correlate gene expression profiles in epileptic rats with epilepsy patients and demonstrate the clinical relevance of our findings, we performed gene analysis on four patient samples obtained from temporal lobectomy and compared to four control brains from NICHD Brain Bank. Pro-inflammatory gene expressions were at higher magnitudes and more sustained in P30. The inflammatory response was driven by the cytokines IL-1β, IL-6, and IL-18 in the acute period up to 72 h and by IL-18 in the subacute period through the 10-day time point. In addition, a panoply of other immune system genes was upregulated, including chemokines, glia markers and adhesion molecules. Genes associated with the mitogen activated protein kinase (MAPK) pathways comprised the largest functional group identified. Through the integration of multiple ontological databases, we analyzed genes belonging to 13 separate pathways linked to Classical MAPK ERK, as well as stress activated protein kinases (SAPKs) p38 and JNK. Interestingly, genes belonging to the Classical MAPK pathways were mostly transiently activated within the first 24 h, while genes in the SAPK pathways had divergent time courses of expression, showing sustained activation only in P30. Genes in P30 also had different regulatory functions than in P15: P30 animals showed marked increases in positive regulators of transcription, of signaling pathways as well as of MAPKKK cascades. Many of the same inflammation-related genes as in epileptic rats were significantly upregulated in human hippocampus, higher than in lateral temporal neocortex. They included glia-associated genes, cytokines, chemokines and adhesion molecules and MAPK pathway genes. Uniquely expressed in human hippocampus were adaptive immune system genes including immune receptors CDs and MHC II HLAs. In the brain, many immune molecules have additional roles in synaptic plasticity and the promotion of neurite outgrowth. We propose that persistent changes in inflammatory gene expression after SE leads not only to structural damage but also to aberrant synaptogenesis that may lead to epileptogenesis. Furthermore, the sustained pattern of inflammatory genes upregulated in the epileptic mature brain was distinct from that of the immature brain that show transient changes and are resistant to cell death and neuropathologic changes. Our data suggest that the epileptogenic process may be a result of failed cellular signaling mechanisms, where insults overwhelm the system beyond a homeostatic threshold.

## 1 Introduction

Epileptogenesis is a theoretical construct whereby concerted cascades of molecular, cellular and network changes precipitate spontaneous recurrent seizures, or epilepsy. Etiology is known for roughly half of diagnosed epilepsies; these symptomatic or acquired epilepsies are often triggered by brain injury (Hauser and Hesdorffer, 1990; Hauser, 1997; Engel, 2006). Up to 53% of patients who experience severe traumatic brain injury (TBI) and between 2% and 5% of patients who experience ischemic stroke will develop epilepsy (Salazar et al., 1985; Menon and Shorvon, 2009). Additionally, epilepsy develops in up to 43% of patients who experience refractory status epilepticus (SE), a prolonged seizure lasting over 30 min (Hesdorffer et al., 1998). Once seizures begin, epilepsy can worsen over time and are frequently accompanied by declining cognitive function (Engel and International League Against Epilepsy, 2001; Briellmann et al., 2002; Sayin et al., 2004; Thompson and Duncan, 2005; Engel, 2006).

Mesial temporal lobe epilepsy (MTLE) is the most common type of focal epilepsy and is often resistant to currently available pharmacotherapy (Loscher, 2002). One of the primary identified risk factors for developing MTLE in adulthood is prolonged febrile seizures in childhood (Baulac et al., 2004). The pathologic process that occurs between the initial insult and the development of frank epilepsy is of great interest in designing therapeutic strategies to prevent epileptogenesis.

A number of hippocampal changes have been proposed to lead to epileptogenesis. One of the classic neuropathologic changes is mossy fiber sprouting from dentate granule cells (Blumcke et al., 1999). Many of these fibers display an aberrant growth pattern that leads them back into the inner molecular layer of the dentate gyrus instead of the CA3 area of the hippocampus, forming recurrent excitatory loops (Tauck and Nadler, 1985; Sutula et al., 1989; Babb et al., 1991). MTLE also displays a specific pattern of neuronal loss in the hippocampal subfields and a dense gliosis that has led to the description of this pathologic pattern as hippocampal sclerosis (Blumcke et al., 2007). In animal models, excitatory interneurons in the dentate gyrus seem to be particularly vulnerable to cell death (Sloviter, 1987). These neurons normally project onto inhibitory interneurons and their loss may therefore lead to decreased inhibition. Furthermore, seizure activity in animal models leads to increased mitotic activity, producing new dentate granule cells which may become abnormally integrated into neuronal circuit (Parent et al., 1997; Scharfman et al., 2000). On a molecular level, there are also changes in the expression and components of different receptors, such as γ-aminobutyric acid A (GABAA) (Brooks-Kayal et al., 1998).

The systemic administration of kainic acid (KA), a glutamate agonist, is frequently used in animal models of MTLE. KA leads to prolonged seizures, status epilepticus, and to the generation of spontaneous seizures after a latent period. These seizures originate from the hippocampus in a similar manner to MTLE (Tremblay et al., 1984). Seizure activity has been shown to alter the expression of a variety of genes for neurotransmitters, ion channels, receptors, transcription factors, intracellular messengers, inflammatory mediators, and neurotrophic factors (Jamali et al., 2006). These changes in gene expression may lead to the pathologic phenomena observed in MTLE. In order to link neuronal activity during seizures with the long-term changes which lead to epilepsy, focus has been primarily on the mechanisms of synaptic plasticity and inflammation.

An increasing amount of evidence indicates that seizures result in activation of the inflammatory responses (Oprica et al., 2003; Vezzani and Granata, 2005). Recent work implicates innate as well as adaptive immunity in the generation of this inflammatory responses (Ravizza et al., 2008). Seizures cause marked glial activation similar to that proposed to contribute to the neuronal damage observed in Alzheimer’s disease (Mrak and Griffin, 2005). The neuroinflammation seen in response to seizures may result in cell death as well as more subtle changes that lead to epileptogenesis. The KA model of MTLE elicits multi-faceted immune responses. KA-induced status epilepticus (KA-SE) causes prolonged astrocyte activation and a microglial activation of shorter duration (Somera-Molina et al., 2007). A strong pattern of astrogliosis has also been classically demonstrated in human MTLE, while animal models show a variety of functional changes in astrocytes (Binder and Steinhauser, 2006). Seizures seems to affect the expression of a wide variety of inflammatory cytokines, particularly IL-1β, chemokines, adhesion molecules, extracellular proteinases, complement components, immune receptors, heat shock proteins, and other immune regulatory molecules (De Simoni et al., 2000; van Gassen et al., 2008).

Reflecting the wide range of functional and anatomical changes observed during epileptogenesis, numerous studies examining the gene expression profiles in epilepsy and its models have previously identified an overwhelming array of transcriptional changes (Jamali et al., 2006; Lukasiuk et al., 2006; Wang et al., 2010; Dixit et al., 2016; Pfisterer et al., 2020). Alongside mechanisms of inflammation and synaptic plasticity, some of these studies have drawn attention to mitogen activated protein kinase (MAPK) activity and MAPK signaling pathways (Okamoto et al., 2010; Hansen et al., 2014; Dixit et al., 2016; Salman et al., 2017; Kalozoumi et al., 2018; Fu et al., 2020; Bencurova et al., 2021). These ubiquitous and highly conserved molecules have evolved to transduce environmental and developmental signals into a wide range of programmed and adaptive cellular responses (Zhang et al., 2002; Arthur and Ley, 2013; Peti and Page, 2013). As MAPK pathways transduce extracellular signals into cellular modifications by means of transcriptional (Whitmarsh, 2007), translational (Kelleher et al., 2004) and epigenetic control (Suganuma and Workman, 2012), misregulation could ostensibly underlie maladaptive cellular responses observed during epileptogenesis.

Our first goal was to identify main categories of genes and their important constituents that contribute to the inflammatory responses in an experimental model of MTLE. By comparing the gene expression profile in tissue samples of human brain removed for intractable MTLE with that of the KA rat model of the disease, we aimed to demonstrate a similar pathologic process and highlight the relevance of animal data to human disease. Second, we aimed to elucidate the temporal pattern of immune gene expression following KA-SE in rats at P15 and P30. While both P15 and P30 animals experience acute SE in response to KA injection, only the P30 animals develop spontaneous recurrent seizures. P15 animals show no cell death and no spontaneous recurrent seizures while P30 animals develop chronic epileptic state (Albala et al., 1984; Stafstrom et al., 1992; Williams et al., 2009; Mlsna & Koh, 2013). Studying the age-dependent changes following KA-SE, therefore, offers a strategy for understanding pathogenesis of epilepsy. By comparing time and age-dependent gene expression in P15 and P30 rats, we sought to differentiate pathological from protective consequences of KA-SE and gain insight into the process of epileptogenesis. Third, we investigated the time course for the expression of various functional groups of genes concentrating on pathway interactions and the implications for pathogenesis of epilepsy. Additionally, we confirmed the validity of microarray gene profiling results by real-time (quantitative) reverse transcriptase polymerase chain reaction (qRT-PCR) and immunohistochemistry and assessed the translational significance of our findings through microarray analysis and qRT-PCR of hippocampal tissue collected from patients diagnosed with MTLE.

## 2 Materials and methods

### 2.1 Seizure induction

Intraperitoneal (IP) injections of KA dissolved in phosphate-buffered saline (PBS) were administered to P15 (3 mg/kg) and P30 (10 mg/kg) Long-Evans male rats (Wilson et al., 2005). Age-specific doses of KA have been determined previously to result in <25% mortality while inducing acute seizures in >60% of animals. Control littermates received equal IP volume of PBS. Only animals with nearly continuous seizures for more than 30 min (KA-SE) were included in the study. P15 is considered roughly equivalent to human infancy/early childhood (Romijn et al., 1991; Avishai-Eliner et al., 2002; Haut et al., 2004; Baek et al., 2016). Likewise, P30 animals were chosen because their response to KA injection is adult-like with neuronal injury and the occurrence of spontaneous seizures. However, P30 might be more accurately considered pubescence rather than adulthood (Nitecka et al., 1984; Stafstrom et al., 1992).

### 2.2 Selection of patients and controls

From our database of surgical pathology samples, we identified four patients who received surgical treatment for MTLE between January 2006 and February 2008 at Children’s Memorial Hospital and Northwestern Memorial Hospital in Chicago (Table 1). These patients all had intractable seizures despite best medical therapy. Seizure semiology consisted of focal impaired awareness seizures and bilateral secondarily generalized tonic-clonic seizures. The diagnosis of MTLE was confirmed by neuropathology and all patients received a full pre-operative workup including magnetic resonance imaging (MRI), electroencephalogram (EEG) or video EEG, and neuropsychiatry testing. Tissue samples of the hippocampus and temporal lobe neocortex were extracted from the anterior temporal lobectomy surgical specimens. The sample from one patient did not contain enough hippocampal tissue for qRT-PCR, leaving only three hippocampal tissues.

**TABLE 1:** Clinical information of temporal lobe epilepsy cases.

Patient ID	Tissue analyzed	Sex	Age at operation (years)	Age of seizure onset (years)	History of febrile seizures	Family history of epilepsy
A14	Neocortex	F	9	7	Yes	No
A16	Neocortex, Hipp	F	68	42	Yes	Yes—2 children
A17	Neocortex, Hipp	M	39	7	No	No
A21	Neocortex, Hipp	M	34	1.5	No	Yes—Maternal uncle

ID, identification; Hipp, hippocampus.

The control group was selected based on cause of death and post-mortem interval (Table 2). The cause of death in all cases was an acute, non-infectious, extracranial process. In order to ensure good ribonucleic acid (RNA) quality, we used tissue only from subjects with post-mortem intervals less than 10 h. Four control tissue samples were obtained from the National Institute of Child Health and Human Development (NICHD) Brain and Tissue Bank for Developmental Disorders at the University of Maryland, Baltimore. Tissues were collected and prepared according to the protocols described on the NICHD Brain and Tissue Bank website.

**TABLE 2:** Clinical information of control cases.

Patient ID	Age at death (years)	Sex	Cause of death	Post-mortem interval (hours)
C1	15	F	Chest injuries—MVA	5
C2	13	M	Asphyxia	5
C3	8	M	Cardiac arrythmia	6
C4	32	F	Seroquel/alcohol intoxication	5

ID, identification; MVA, motor vehicle accident.

### 2.3 Hippocampal dissection and preparation of RNA

Rats were sacrificed at 1, 6, 24, 72, and 240 h after seizure induction (*n* = 12/time point per age group). Animals were deeply anesthetized with isoflurane, decapitated, and the brains were removed. Both hippocampi from each animal were rapidly dissected, frozen in isopentane cooled with dry ice, and stored at –80°C. Individual hippocampal tissues (0.15–0.2 g) were homogenized in Trizol reagent^®^ (Invitrogen, Carlsbad, CA, United States) using a glass homogenizer (Wheaton Industries, Millville, NJ, United States), and total RNA was isolated following the manufacturer’s protocol. RNA concentration and purity was determined spectrophotometrically by Gene QuantPro^®^ (Amersham Biosciences GE, Piscataway, NJ, United States). For microarray analysis, equimolar quantities of RNAs were pooled from four animals for each sample. Individual RNA sample for qRT-PCR was treated with RNase-free DNase I (Roche Diagnostics, Indianapolis, IN, United States) for 20 min at room temperature, followed by inactivation for 10 min at 75°C. All of the RNA samples for microarray and qRT-PCR were further purified using RNA Easy Kit (Qiagen, Valencia, CA, United States), according to the manufacturer’s instructions, in order to remove any remaining genomic DNA and salts.

Human samples were collected intra-operatively. The hippocampus and temporal neocortex were immediately dissected and frozen in isopentane cooled with dry ice. RNA was extracted and prepared using the same technique described above with the sole difference that RNA for microarray studies consisted of samples from individual patients rather than pooled RNA from several animals. For each MTLE patient, temporal neocortex and hippocampal samples were processed separately.

### 2.4 Microarray analysis

For the rat microarray experiments, we used the RG34A high-density oligonucleotide Affymetrix Genechip arrays. Three independent hybridizations were performed per condition: Control, KA; 1, 6, 24, 72, and 240 h; P15, P30, total of 60 profiles. Preparation of cRNA, array hybridization, and scanning were performed by Microarray Consortium (NINDS/NIMH) at TGEN (Phoenix, AZ). Affymetrix Microarray Suite (v. 5.0) was used for probe-level analysis. The analysis relies on the interpretation of probe set hybridization performance (pairs of 16 perfect match and mismatch 25-mer oligonucleotides per probe set) as a measure of whether signal intensities are significantly above background and specific to the gene of interest. A signal value is produced that represents the relative level of expression of a transcript. Additionally, the detection algorithm uses probe pair intensities and assigns a present, marginal, or absent call, after comparing with a predefined threshold.

We included in our data analysis only those probe sets that were present in at least two out of three samples in either control or KA. In each time point, the selected genes are >4,000 out of 8,799. This stringent threshold effectively eliminated genes with low precision or those with expression levels too close to the background. We used GeneSpring (v. 5.03) for normalizing data, selecting genes for fold change and performing statistical group comparison between control and KA to generate the *p* value. We also used Significance Analysis of Microarrays (SAM v. 1.21, Stanford University) for additional statistical analysis, which generates a q value that reflects the false discovery rate (Storey and Tibshirani, 2003). Select genes are considered significant if *p* < 0.05 and *q* < 0.05 in at least one time point.

For the human microarray experiments, we used the Human Genome U133 Plus 2.0 Array. For each patient, individual hybridizations were performed for each tissue type, hippocampus and temporal neocortex. Three hippocampal MTLE tissues, four temporal cortex MTLE tissues, and four control neocortical tissues were analyzed. The preparation of cRNA, array hybridization, and scanning were performed by the Microarray Consortium at TGEN as described above.

The same probe-level analysis was applied to human data. However, due to the fact that human arrays contain more probe sets and that more sensitive algorithm was used for detecting lower signal intensities, we included in our data analysis only those probe sets that were present in all samples. Still, more than 20,000 probe sets (out of 54,675) passed the criteria in each comparison against control. The genes were selected both by fold change (1.5) and by *t*-test (*p* < 0.05).

### 2.5 Real time reverse transcriptase polymerase chain reaction

qRT-PCR was used for validation of both rat and human microarray results. The levels of gene expression of 84 inflammatory factors and MAPK related genes [including MAPK kinase (MAPKK) and MAP kinase kinase (MKK)], five housekeeping genes, three positive controls, and three reverse transcription controls were measured using the RT2Profiler™PCRArray (SuperArray Bioscience Corp., Frederick, MD, United States). In addition to genes related to the MAPK signaling pathway, this array profiles the expression of transcription factors, Raf regulating proteins, MEK kinase 1 (MEKK1) interacting proteins, cell cycle proteins regulated by the extracellular-signal regulated kinase (ERK) 1/2 pathway, and genes whose expression is induced by MAPK.

RT2Profiler™PCRArray is a qRT-PCR based and performed in a 96-well plate. The RNA samples from animals and patients were placed into individual RT2Profiler™PCRArrays. First, 1 µg of each RNA sample was converted to first strand cDNAs using the RT2 First Strand Kit following the manufacturer’s user manual. The cDNA was added to the RT2 qPCR Master Mixes containing SYBR Green. The mixtures were aliquoted into 96-well plate containing pretreated gene specific primer sets. The PCR reactions were performed using ABI Prism 7,500 system (Applied Biosystems, Foster City, CA, United States) with the following parameters; cycle 1 (1 repeat), 10 min at 95°C; and cycle 2 (40 repeats), 15 s at 95°C and followed by 1 min at 60°C, and the data were collected at 60°C.

The cycle threshold values (CT) for genes of interest were first normalized to that of housekeeping genes, β-actin and/or GAPDH, in the same sample and expressed as percentage of controls. This normalized Ct (ΔCt) was used to compare control vs. treated samples (ΔΔCt), which is then expressed as fold change of gene amplification. The Student’s t-test (unpaired) was used with the standard significance level of *p* < 0.05. Analysis was performed using GraphPad Prism 4 (GraphPad Software, LaJolla, CA, United States).

### 2.6 Database integration and classification of genes

Several gene ontology databases were used to investigate the functional importance of differentially expressed genes. Functional analysis was performed in Mathematica 6.0.1 and Mathematica 7.0.0 (Wolfram Research, Champaign, IL, United States) using pattern matching capabilities and nine online databases: Affymetrix NetAffxTM at www.affymetrix.com/analysis/index.affx (Liu et al., 2003), GeneCards—human only at www.genecards.org (Safran et al., 2002; Stelzer et al., 2008), NCBI Entrez Gene at http://www.ncbi.nlm.nih.gov/gene/ (Maglott et al., 2005), KEGG pathway at http://www.genome.jp/kegg/pathway.html (Kanehisa et al., 2002), Gene Ontology at http://www.geneontology.org/ (Ashburner et al., 2000), AmiGO at http://amigo.geneontology.org/cgi-bin/amigo/go.cgi, the Rat Genome Database at http://rgd.mcw.edu/ (Twigger et al., 2007), Panther at http://www.pantherdb.org/ (Thomas et al., 2003), and the Pathway Interactions Database at http://pid.nci.nih.gov/ (Schaefer et al., 2009).

After retrieving the database information, Mathematica was used to create a composite functional and pathway-related database for significantly expressed genes and their aliases. Human gene aliases and homologues were identified primarily using GeneCards and secondarily using the Rat Genome Database and NCBI Entrez Gene. Mathematica’s pattern matching capabilities were then used to identify functional and pathway groupings of the genes involved.

The composite database was created with code that searched through various database files by gene name and alias and integrated all unique ontological descriptions for each gene across databases. Important functional and pathway ontologies were identified by two consecutive methods: first, consistently reappearing ontological terms were identified and the occurrence of each ontological term in the composite database was then counted. Functional and pathway-related gene groupings were then created with code that searched through the ontological descriptions by user-defined key words (i.e. “inflammation,” “inflammatory response”).

The gene expression data analyzed through GeneSpring and SAM was then transferred into Mathematica. Mathematica then reorganized the data by group (P15, P30, Controls), gene name, time-point of expression, and functionality. Programming code also generated line graphs and tables to reflect the data. Line graphs of gene expression levels were fitted using second order interpolation for ease of visualization.

### 2.7 Pathway analysis

Initial results and data analysis pointed to a significant role of MAPKs in KA-SE. Consequently, pathways involving MAPK genes were analyzed from several perspectives. MAPK pathways have been described as continuously variable switches dependent on various factors with multiple upstream and downstream pathways feeding into the primary MAPKs: ERK, p38 and Jun N-terminal kinase (JNK). A single gene may be implicated in multiple pathways, therefore one association may not be more “correct” than the other. We picked our pathways in the spirit of “modular biology” (Hartwell et al., 1999; Bruggeman et al., 2002; Ingolia and Murray, 2007), still acknowledging that canonical representations of discrete and linear signaling transduction systems may not be suitable models, especially in response to highly deleterious stimuli.

We analyzed MAPK related cascades into 13 component pathways: ERK, Growth Factor Signaling, Ras/Rab, p38, Rho/Rac/Cdc42, JNK, p53, TGF-β, TNF-α, JAK/STAT, Wnt, NF-κB, and PI3K/AKT. Although genes differentially expressed in P15 or P30 were annotated with numerous pathways, only pathways with uniquely expressed genes were selected. Each gene was assigned a pathway set—i.e., according to the databases, a gene was associated with the regulation and/or transduction of one or more of the 13 component pathways. Single-pathway associated genes were organized and analyzed separately from multiple-pathway associated genes. Genes classified as transcription factors and/or co-factors or transcriptional regulators were analyzed as well. Finally, genes associated with regulation of pathways, MAPKKKs, phosphatase, and kinase activity were identified and analyzed.

### 2.8 Immunohistochemistry

Immunohistochemistry was performed on horizontal sections of rat brain using antibodies to CD74 or Hsp70 (DAKO, Glostrup, Denmark). Sections were mounted on premium microscope slides (Fisher Scientific, Pittsburgh, PA), fixed in paraformaldehyde and incubated with anti-CD74 or anti-Hsp70 at 4°C overnight, followed by a biotinylated secondary anti-IgG, using a previously published method (Koh et al., 1999). The sections were then treated with HRP-conjugated streptavidin. Specimens were then examined under light microscopy.

## 3 Results

### 3.1 Higher magnitude and longer duration of gene expression in response to KA-SE at P30

**FIGURE 1:**
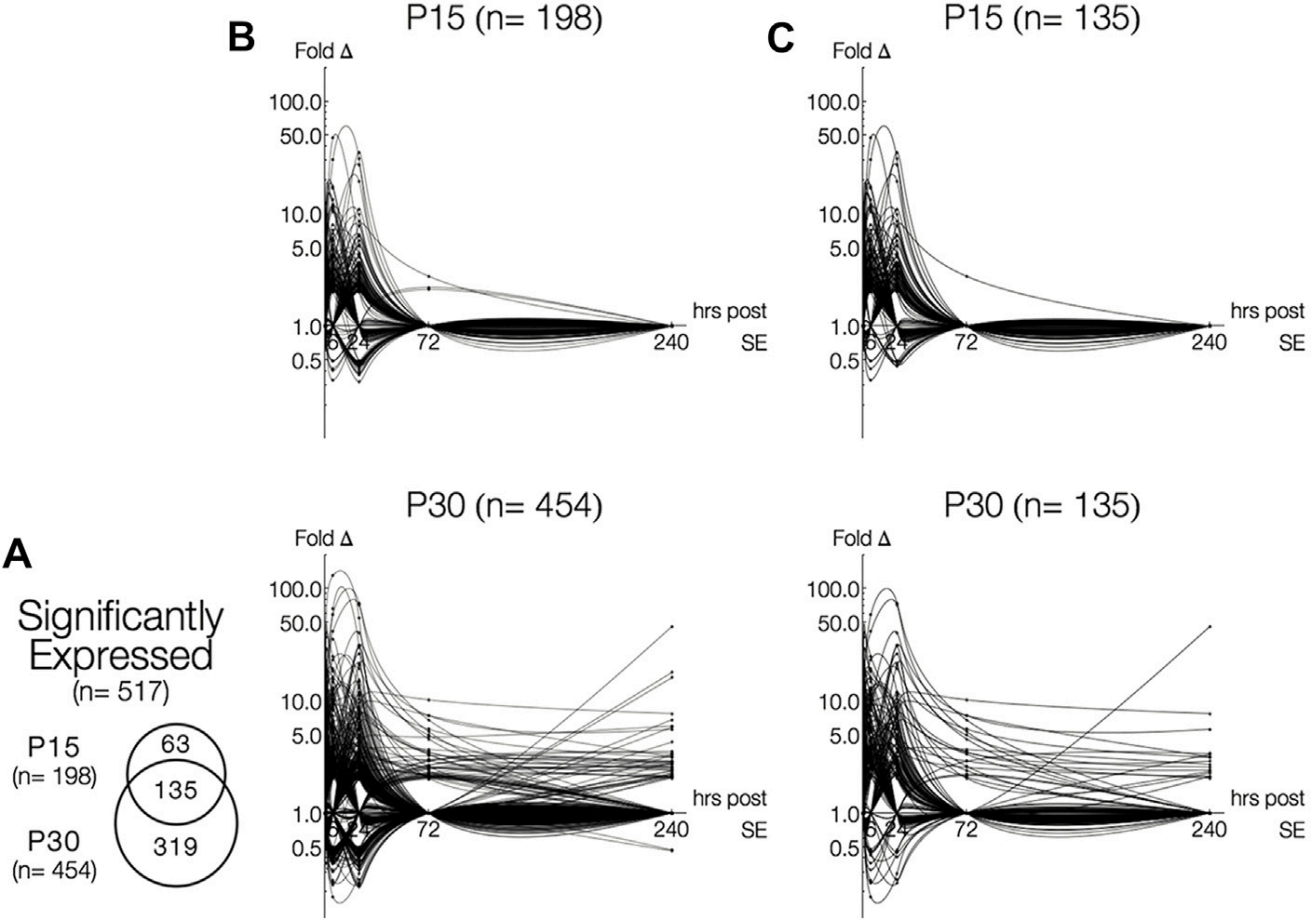
Time-and age-dependent gene regulation in rat hippocampus after KA-SE. **(A)** Venn Diagrams of all differentially expressed genes (fold change > 2, *p* < 0.05, q < 0.05). While majority of genes (135/198, 68%) in P15 are also expressed in P30, many genes (319/454, 70%) are expressed only in P30. **(B)** Log-linear plots of differential gene expression (fold change) of all significant genes 1–240 h after KA. Data points are indicated by black circles. Lines, each representing a single gene, were fitted using second order interpolation for ease of visualization. **(C)** Log-linear plots of differential gene expression of common genes between P15 and P30.

A total of 517 genes were differentially expressed (>2-fold change, *p* < 0.05, q > 0.05 or <0.5-fold change, *p* < 0.05, q > 0.05) in P15 (*n* = 198) and P30 (*n* = 454) hippocampi at 1, 6, 24, 72 and 240 h after seizure induction compared to PBS injected control littermates (Figure 1A). More genes were differentially expressed at higher magnitudes in P30 compared P15 (Figure 1B); 135 genes were commonly expressed in P15 and P30 (Figure 1C). Differential gene expression remained below 50-fold change in P15 while it exceeded 100-fold in P30. By 72 h, only three genes (S100a4, Ccnc, Kcna4) were above baseline in P15, while marked gene upregulation persisted in P30 (*n* = 48, 72 h; *n* = 43, 240 h) throughout the 10 days. At each time point, more genes were upregulated in P30 (*n* = 57, 1 h; *n* = 155, 6 h; *n* = 196, 24 h; *n* = 48, 72 h; *n* = 43, 240 h) than P15 (*n* = 31, 1 h; *n* = 99, 6 h; *n* = 92, 24 h; *n* = 3, 72 h).

Although many genes were upregulated at only a single time point in both age groups (*n* = 118, P15; *n* = 186, P30), more genes were upregulated for a longer time in P30 compared to P15. In P15, only five genes have extended time-courses of upregulation: Ania-2, Ania-4, Bdnf and Nptx2 were upregulated 1–24 h, and S100a4 was upregulated at 24 and 72 h. In P30, 41 genes were upregulated for an extended time (3 or more time points) and 28 genes were upregulated at both 24 and 72 h. Remarkably, Mt2a, Hspb1, Lgals3, Lox, and Spp1 are all upregulated 6–240 h.

A similar pattern was observed in downregulated genes (*n* = 31, P15; *n* = 137, P30). In P15, downregulation took place only at 6 (*n* = 6) and 24 h (*n* = 25), while downregulation in P30 occurred at 1, 6, 24, and 240 h (*n* = 4, 1 h; *n* = 71, 6 h; *n* = 71, 24 h; *n* = 2, 240 h). In P15, all genes were downregulated for only a single time point. For P30 animals, nine genes (Adra1d, Dbp, Dcn, Gucy1a, Hnmt, Htr5b, Kit, Neurod1, Pk1b) were downregulated at 6 and 24 h.

### 3.2 Microarray findings of inflammatory response

**FIGURE 2:**
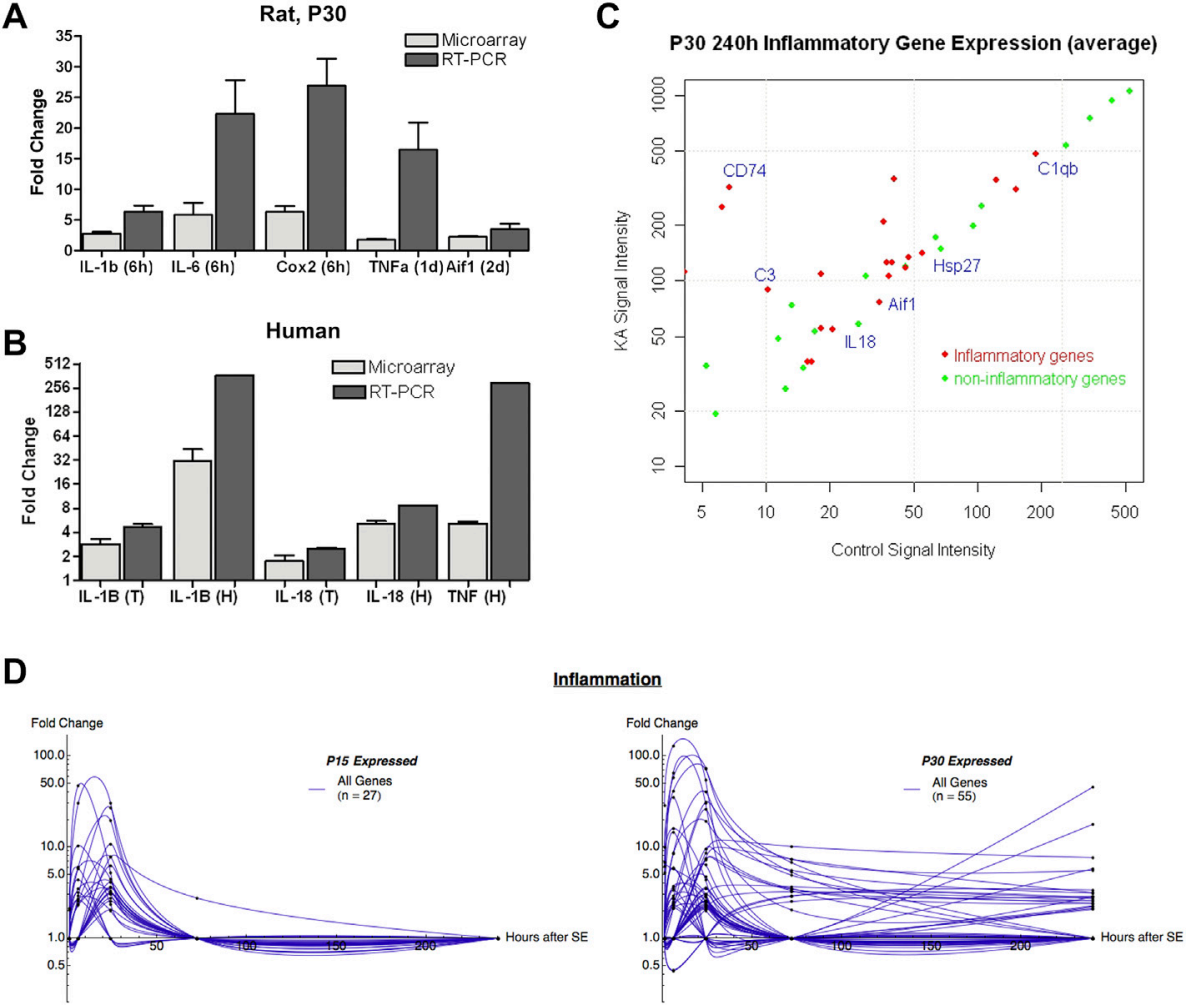
Time- and age-dependent expression of inflammatory genes after KA-SE: qRT-PCR validation and human data correlation. **(A)** Fold change in expression in select inflammatory genes: comparison between the microarray and qRT-PCR results in rats at P30. **(B)** Fold change in expression in select inflammatory genes: comparison between the microarray and quantitative RT-PCR results in human. T, temporal lobe; H, hippocampus. **(C)** Inflammatory genes comprised a significant proportion of persistently upregulated genes at 240 h after KA-SE in P30. Red dots represent inflammatory genes. Green dots represent non-inflammatory genes. **(D)** Summary graph of the time course of fold change in inflammatory genes. Note the inflammatory genes remain significantly elevated at 240 h only in P30 rats.

Figure 2A compares fold change in expression between the microarray and quantitative RT-PCR results among a few representative genes in rats at P30. The relative expression levels between the various genes are preserved between microarray and RT-PCR. Similarly, Figure 2B compares fold changes in expression for a selected group of genes between the same two methods of amplification. Gene expression is shown for both temporal neocortex and hippocampal tissues. Fold changes were often higher in the qRT-PCR than in microarray, likely reflecting a greater linear dynamic range afforded by qRT-PCR. Marked differences in fold change between microarray and RT-PCR may also be due to sampling differences. Each value in microarray represents an average of 12 animals (pooled RNAs from four animals, three independent hybridizations) whereas only an individual sample was run in qRT-PCR (RNA samples were not pooled).

Figure 2C shows all genes significantly upregulated in the P30 animals at 10 days after KA-SE. A large proportion of upregulated genes at this time point are inflammatory in nature and many of these are expressed at high levels, highlighting the importance of the immune response in the brain’s response to seizures.

In order to compare the seizure-induced inflammatory response in P15 with that of P30, all inflammatory genes were graphed over time (Figure 2D). Many more genes are upregulated to significant levels in P30 compared to P15. Furthermore, P30 also shows more prolonged and higher levels of expression of inflammatory genes.

### 3.3 Time and age dependent regulation of inflammatory markers

#### 3.3.1 Microglial and astrocyte activation

**FIGURE 3:**
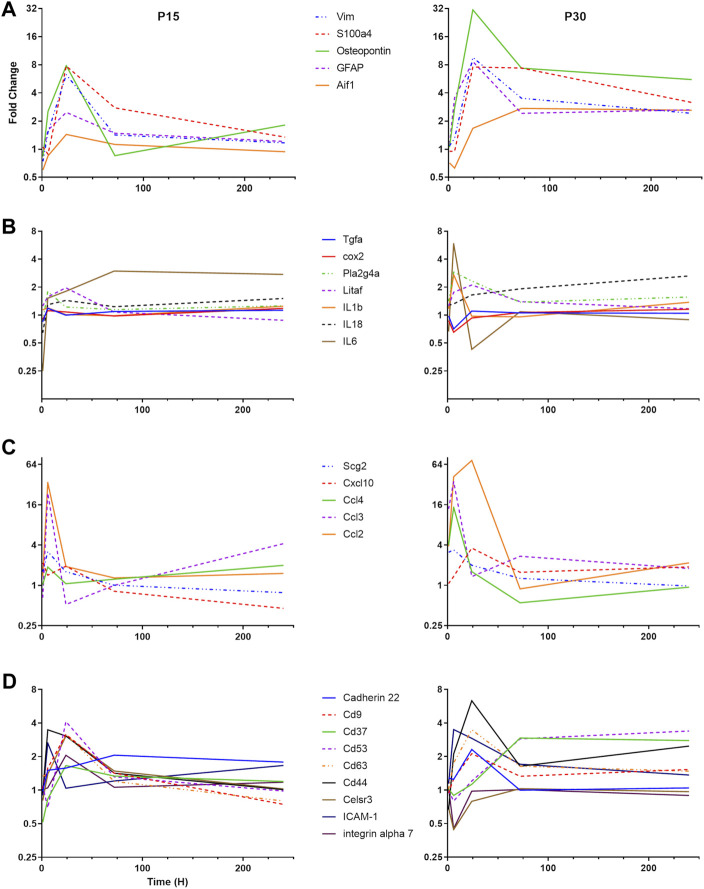
Differential time course of various groups of inflammatory genes in P15 and P30. **(A)** Microglial and astrocyte markers (Aif1, GFAP, Osteopontin, S100a4, and VIM). **(B)** Cytokines and eicosanoid pathways (IL-1β, IL-6, IL-18, LITAF, TGF-β R1, TNF R1, PLA2G4A, and COX2). **(C)** Chemokines (CCL2, CCL3, CCL4, CXCL10, and Scg2). **(D)** Adhesion molecules (Cadherin 22, CD9, CD37, CD 53, CD63, CD44, Celsr3, ICAM-1, and Integrin α7).

To understand the differential pattern of activation among different CNS cell types, we focused our attention to cell-specific markers (Figure 3A). GFAP and vimentin are intermediate filaments that have traditionally been used to investigate astrocyte activation (Eng and Ghirnikar, 1994). The S100 group of proteins bind calcium and are involved in a large number of cellular functions. S100B is classically used as an astrocyte marker but S100a4 has been found to be involved in cellular migration and is upregulated specifically in white matter astrocytes after CNS injury (Kozlova and Lukanidin, 2002). We found that astrocyte activation in P30 rats peaked at 24 h after KA-SE and remained elevated through the 10-day time point. In contrast, P15 rats have lower levels of astrocyte activation at all time points and the upregulation of astrocyte markers was largely complete by 72 h.

Aif-1 is a marker of microglial activation, which is upregulated in response to CNS injury (Schwab et al., 2001). Osteopontin (Spp-1) is secreted by a subpopulation of activated microglia in response to a variety of insults, including stroke and systemic KA administration (Ellison et al., 1998; Kim et al., 2002). In the P15 animals, Spp-1 expression is only significantly upregulated at the 24 h time point, while Aif-1 is not significantly upregulated at any time point. In the P30 animals, Spp-1 upregulation peaks at 24 h after KA-SE but is significantly upregulated from 6 to 240 h. Aif-1 upregulation in P30 animals is delayed, with increased expression only at 72 and 240 h.

#### 3.3.2 Cytokines and eicosanoid pathways

Cytokine genes are significantly elevated only in the P30 animals (Figure 3B). IL-1β and IL-6 gene expression are increased at 6 h after KA-SE. The IL-18 gene is upregulated in a biphasic manner, with significant increase at 24 and 240 h after KA-SE. Lipopolysaccharide-induced TNF factor, LITAF, is a transcription factor for TNF-α. It is upregulated to modest levels at 24 h. The TNF receptor type 1 and the TGF-β receptor type 1 are also upregulated to modest levels in the P30 animals at 24 h after KA-SE.

Eicosanoid production begins with the conversion of phospholipids or diacylglycerol into arachidonic acid by Phospholipase A2. Downstream, COX-2 catalyzes the conversion of arachidonic acid into an intermediate, which results in the production of Prostacyclin, Thromboxane A2 and a variety of prostaglandins. We saw upregulation of PLA2G4A, a cytosolic phospholipase A2 isozyme, at 6 and 24 h only in P30 rats. However, COX2 is upregulated in both P15 and P30 animals. In the P15 group, COX2 expression is increased 1 and 6 h after KA-SE while in the P30 group, COX2 expression is of higher intensity and longer duration, peaking at 1 h but persisting through 24 h.

#### 3.3.3 Chemokines

There was a dramatic upregulation of chemokine expression in both P15 and P30 (Figure 3C). In P15 rats, Ccl2 is only significantly upregulated at the 6 and 24 h time points, peaking at 6 h. However, in P30 rats it is upregulated at all but 240 h, peaking at 24 h. Cxcl10, Ccl3 and Ccl4 are upregulated in the P30 animals at 6 h after KA-SE. The product of the gene Scg2 is a neuropeptide which can be modified to produce secretoneurin. Scg2 is upregulated only at 6 h after KA-SE in P15 animals, while in P30 animals Scg2 is upregulated at both 1 and 6 h.

#### 3.3.4 Adhesion molecules

The tetraspanins are a large family of scaffolding membrane proteins that function in cell adhesion, motility, activation, and proliferation (Figure 3D). This group includes CD9, CD37, CD53, and CD63. In P15 rats, the tetraspanins CD9, CD53, and CD63 are upregulated at 24 h. P30 animals also show an increase in the expression of CD9 and CD62 at 24 h after KA-SE followed by a late upregulation of CD37 and CD53 at 72 and 240 h after KA-SE.

Cadherin 22, an adhesion molecule which is primarily expressed in brain tissue, is only upregulated in the P30 group at 24 h. Celsr3 is a seven-pass transmembrane cadherin primarily involved in neuron-neuron adhesion and the inhibition of neurite outgrowth (Shima et al., 2007). This molecule, along with integrin α7, show an interesting pattern of expression. They are upregulated at 24 h in P15 animals while downregulated at 6 h after KA-SE in the P30 group.

Other adhesion molecules are also known to function in leukocyte recruitment and extravasation. ICAM-1 is transiently expressed in the P15 group only at 6 h. However, in the P30 group, ICAM-1 showed a prolonged upregulation, reaching significance at 6 and 24 h. CD44 expression is increased to significant levels in both P15 and P30 animals at the 6 and 24 h time points but it reaches much higher levels of expression in P30 animals at 24 h after KA-SE.

### 3.4 Mesial temporal lobe epilepsy in humans

**FIGURE 4:**
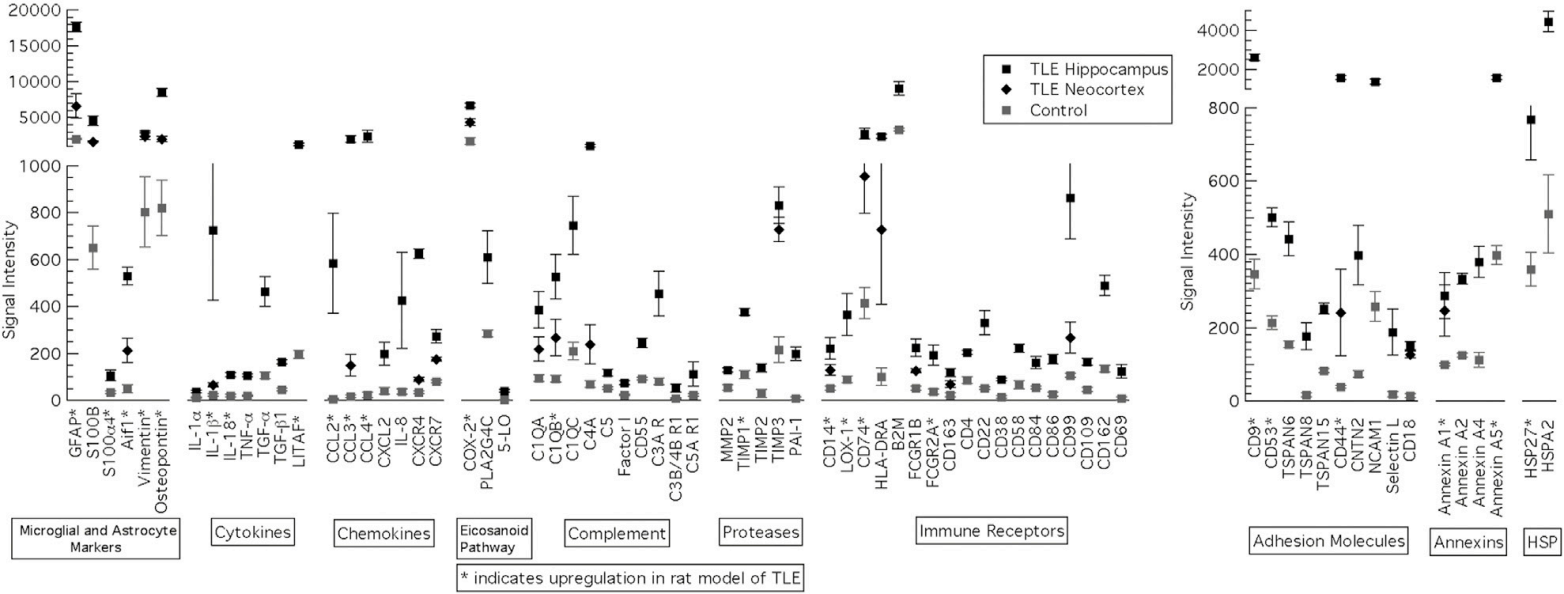
Genes significantly upregulated in the hippocampus and lateral temporal neocortex of patients with MTLE. Inflammatory genes were classified into 10 groups: microglia and astrocyte markers, Cytokines, Chemokines, Eicsanoid Pathway, Complement, Proteases, Immune Receptors, Adhesion Molecules, Annexins and heat shock proteins (HSP). *Genes also upregulated in the rat KA model of MTLE. Reproduced with the permission from the author (SK), J Child Neurology 2018. vol 33 (1) 64–72.

Several genes were significantly upregulated in the hippocampus and lateral temporal neocortex of patients with MTLE (Figure 4). Many of the same genes are upregulated in the KA-SE rat model of MTLE and hippocampal tissue from patients with MTLE. With the exception of HSPs, the human hippocampus data generally includes a larger number of significantly upregulated genes than the rats within each category. A particularly glaring difference between our human and rat data is the upregulation of a large number of genes involved in the activation of the adaptive immune system only in MTLE patients. This includes immune receptors such as CD4, CD22, CD38, CD58, CD69, CD84, CD86, CD99, CD109, and CD162. Of note, only HLA-DRA is graphed as a member of the MHC II group of gene but we also find significant upregulation of HLA-DQA1/2, HLA-DPA1, HLA-DPB1, HLA-G, HLA-DOA, HLA-C, HLA-DMB, HLA-DRB1/3/4, HLA-DMA, HLA-B, and HLA-A. A smaller subset of genes from each category is also upregulated in the lateral temporal neocortex in addition to the hippocampus. In all these cases, expression in the neocortex was lower than in the hippocampus.

### 3.5 Functional distribution of differentially expressed genes and MAPK signaling pathway: The most abundant functional groups

**FIGURE 5:**
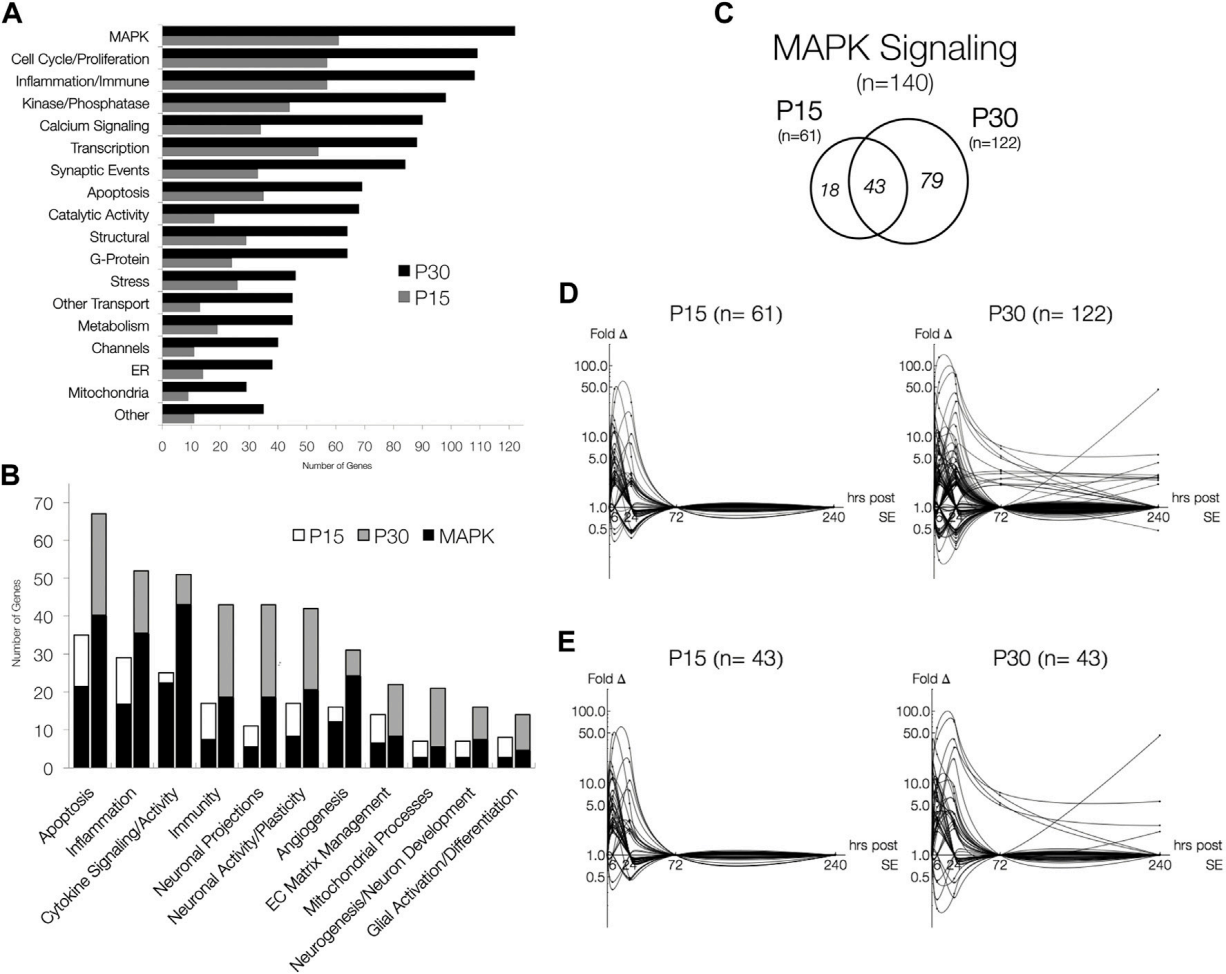
Functional Distribution of Differentially Expressed Genes and MAPK Signaling pathway, the most abundant functional groups. **(A)** Genes involved in processes associated with the latent period and MAPK signaling. Genes were categorized in agreement with databases. **(B)** The number of genes associated with MAPK signaling in P15 and P30 animals across all time points. Significantly expressed genes are more numerous in P30 than in P15, but relative MAPK co-categorization is comparable. MAPK overlaps most with cytokine signaling/activity (24/25, P15; 46/51, P30) and least with mitochondrial processes (3/7, P15; 6/21, P30) and glial activation/differentiation (3/8, P15; 5/14, P30). **(C)** Venn Diagrams of differentially expressed genes belonging to MAPK Signaling (fold change > 2, *p* < 0.05, q < 0.05). While majority (70.5%) of genes in P15 are also expressed in P30, most (64.8%) genes are only expressed in P30. **(D)** Log-linear plots of differential gene expression (fold change) of MAPK genes 1–240 h after KA-SE. Data points are indicated by black circles. Lines, each representing a single gene, were fitted using second order interpolation for ease of visualization. **(E)** Log-linear plots of differential gene expression (fold change) of common MAPK genes between P15 and P30.

After database integration (see Section 2.6), we performed an inclusive ontological survey of all differentially expressed genes in both age groups across all 5 times points (Figure 5A). MAPK signaling (*n* = 61, P15; *n* = 122, P30), comprising a total of 140 differentially expressed genes, was the largest functional group identified. The next largest groups of genes differentially expressed are associated with inflammation, immune responses (*n* = 57, P15; *n* = 108, P30), and cell cycle and proliferation (*n* = 57, P15; *n* = 109, P30). Genes related to transcription (*n* = 54, P15; *n* = 88, P30), synaptic events (*n* = 33, P15; *n* = 84, P30), calcium signaling (*n* = 34, P15; *n* = 90, P30) and apoptosis (*n* = 35, P15; *n* = 69, P30), were also differentially expressed in both P15 and P30 animals. Although none of these groups are as large in MAPK, they have all been previously implicated as potential epileptogenic alterations (Pitkanen et al., 2007; Pitkanen et al., 2009).

We next investigated the overlap between MAPK signaling and putative epileptogenic events such as inflammation, neuronal activity, and glial activation. Within functionally relevant genes, we identified those associated with MAPK signaling (Figure 5B). Although all groups identified are present in both P15 and P30, P30 differentially expressed more genes across all functional groups. Many genes associated with each process are also involved in MAPK signaling.

Figure 5C summarizes the number of genes associated with MAPK signaling in P15 and P30 animals across all time points. Again, more genes were differentially expressed at higher magnitudes for longer time courses after KA in P30 compared to P15 (Figure 5D). All MAPK genes returned to baseline by 72 h in P15 while they remained upregulated (*n* = 13, 72 h; *n* = 10, 240 h) in P30. MAPK genes differentially expressed in both age groups (*n* = 43) were expressed at higher magnitudes over longer time courses in P30 (Figure 5E). Notable examples included: Ccl2 (peak = 47.2 fc, 6 h), Timp1 (peak = 30.6 fc, 24 h), Spp1 (peak = 7.8 fc, 24 h), and Hspb1 (peak = 40.4 fc, 24 h) were all upregulated only at 6 and 24 h in P15 animals. In P30, however, Ccl2 (peak = 74.0 fc, 24 h) and Timp1 (peak = 71.4 fc, 24 h) were upregulated 1–72 h, and Spp1 (peak = 31.0 fc, 24 h) and Hspb1 (peak = 41.3 fc, 24 h) were upregulated 6–240 h. Similarly, Ret (2.88 fc, 6 h) and Cd74 (10.85 fc, 24 h) were both upregulated at single time points in P15, while in P30, Ret (peak = 5.68 fc, 6 h) was upregulated at 6, 24 and 240 h and Cd74 (peak = 45.6 fc, 240 h) was upregulated at 24 and 240 h.

The age-dependent nature of our study introduced a risk that our results were confounded by developmental differences. To account for this possibility, we compared the gene expression profiles of P30 controls (treated with PBS instead of KA) with P15 controls (see Section 2.4). 117 genes were differentially expressed between age groups (fold change >2, *p* < 0.05, q < 0.05) 1–24 h after PBS injection; 35 of these genes were also differentially expressed in either P15 or P30 at 1, 6, 24, 72 or 240 h after KA-SE (data not shown). Amongst the 117 genes differentially expressed between PBS injected P15 and P30, 23 were associated with MAPK signaling. Only 9 of the 23 MAPK genes differentially expressed between P15 and P30 controls were also differentially expressed in response to KA-SE in either P15 or P30. This is in stark contrast to the 140 genes differentially expressed in P15 or P30 after KA-SE.

To investigate how baseline differences in gene expression may affect gene expression after seizure induction, we examined the relative abundance of mRNA for these 9 genes in P15 and P30 controls and compared them to seizure-induced changes in expression in P15 and P30 (Table 3). Changes in gene expression in response to seizure were diverse in both age groups and independent of baseline differences in relative mRNA signal. This suggested that differences in changes observed in MAPK gene expression profiles between P15 and P30 (after KA-SE) were age-dependent differences in seizure response rather than an artifact of development.

**TABLE 3:** Relative baselines of mRNA signal and qualitative changes in gene expression amongst MAPK genes differentially expressed between P15 and P30 controls and differentially expressed in P15 or P30 after kainic acid induced status epilepticus (KA-SE).

Gene	Phosphate-buffered saline	Kainic acid	
	Relative mRNA signal	P15	P30
Bdnf	P15 > P30	Upregulated	Upregulated
Hmgcr	P15 > P30	Upregulated	Upregulated
Gadd45a	P15 > P30	Upregulated	Upregulated
Prkaa2	P15 > P30	Downregulated	
Fkbp1b	P15 > P30		Downregulated
Cat	P15 > P30		Downregulated
Camk2a	P30 > P15		Downregulated
Itpr1	P30 > P15		Downregulated
Pdk1	P30 > P15	Upregulated	

RNA, ribonucleic acid; MAPK, mitogen-activated protein kinase; BDNF, brain-derived neurotrophic factor; HMGCR, 3-hydroxy-3-methylglutaryl-CoA reductase; GADD45A, growth arrest and DNA damage inducible α; PRKAA2, protein kinase AMP-activated catalytic subunit α2; FKBP1B, FKBP prolyl isomerase 1B; CAT, catalase; CAMK2A, calcium/calmodulin dependent protein kinase IIα; ITPR1, inositol 1,4,5-trisphosphate receptor type 1; PDK1, pyruvate dehydrogenase kinases 1; P, postnatal.

### 3.6 Pathway-based distribution of differentially expressed MAPK genes

Genes are annotated in online databases by pathways as well as functions such as “apoptosis.” Annotations include both pathways (e.g. “p53 signaling pathway”) as well as regulatory schemes (e.g. “positive regulation of I-κB kinase/NF-κB cascade”).

Many pathways were associated with the differentially expressed genes found in our study. For simplicity, we reduced MAPK signaling networks into 13 pathways: ERK, Growth Factor Signaling, Ras/Rab, p38, Rho/Cdc42/Rac, JNK, p53, TGF-β, TNF-α, JAK/STAT, Wnt, NF-κB, and PI3K/AKT. These 13 were chosen according to a single criterion: each pathway contained least one unique gene differentially expressed in P15 or P30 after KA-SE (see Section 2.7). Reduction of MAPK signaling into 13 component pathways excluded 6 genes which were not annotated with any specific pathway in the databases. 5 of these genes were differentially expressed in P15 and 5 were differentially expressed in P30. Although these 6 genes were expressed differently in P15 and P30, additional inclusion of these genes (data not shown) did not affect the overall patterns of the data presented below.

A total of 134 genes in P15 (*n* = 56) or P30 (*n* = 117) are annotated with the 13 pathways, and 80 of these genes (*n* = 33, P15; *n* = 69, P30) are annotated with only a single pathway. Even though single-pathway annotated genes constitute the majority of the genes expressed in P15 and P30, the majority of genes annotated within a given pathway (in P15 and P30) are annotated with multiple pathways (Table 4).

**TABLE 4:** Thirteen representative pathways for differentially expressed genes in P15 and P30 after KA-SE.

Pathway	P15		P30	
	All genes	Single pathway	All genes	Single pathway
ERK 1/2	9	3	14	5
Growth Factor	18	9	25	9
Ras/Rab	10	3	19	5
P38	12	1	19	5
Rho/Cdc42/Rac	4	0	11	4
JNK	8	0	16	3
P53	12	3	25	5
TGF-β	17	3	23	6
TNF-α	0	0	8	4
NF-κB	13	5	20	8
Wnt	6	2	22	5
JAK/STAT	5	3	10	5
PI3K/AKT	5	1	13	5

P, postnatal; ERK, extracellular signal-regulated kinase; JNK, Jun NH_2_-terminal kinase; TGF-β, transforming growth factor-β; TNF-α, tumor necrosis factor α; NF-κB, Nuclear factor kappa B; JAK/STAT, janus kinase-signal transducer and activator of transcription; PI3K, phosphoinositide 3-kinases.

Although cellular signaling pathways are traditionally characterized as discrete and separate, the vast amount of crosstalk among them makes separation into causal relationships difficult. Whether crosstalk between pathways is the rule or the exception still remains unclear. Furthermore, an emerging idea of signaling pathways comprising a network, which together is responsible for cellular decisions, is starting to eclipse the prior understanding of discrete, linear, and separate pathways. Against this backdrop, we examined different kinds of associations and considered single-pathway annotated and multiple-pathway annotated genes separately.

### 3.7 Single-pathway associated genes

The most studied MAPK families are ERK, p38 and JNK (Morrison, 2012). While these MAPKs respond to a variety of stimuli and there is a considerable amount of crosstalk between them (Shen et al., 2003; Fey et al., 2012), they initiate exquisitely specific cellular responses and have traditionally been split between the classical (the first discovered) MAPK ERK and the stress activated protein kinases (SAPKs), p38 and JNK. With regard to epileptogenesis, ERK has been associated with synaptic remodeling and plasticity while p38 and JNK tend to be associated with immune and inflammatory responses (Gautam et al., 2021). It should be noted that these delineations are oversimplifications and cellular responses may often involve concerted action across interacting pathways (Liu, 2011; Kyriakis and Avruch, 2012; Gautam et al., 2021). Stress related pathways had a different distribution in P15: there were no TNF-α annotated genes and almost no single-pathway annotated genes for p38, JNK, and Rho/Cdc42/Rac (Table 4). To investigate this further, we grouped the single-pathway annotated genes based on their MAPK families: Classical (ERK1/2, Growth Factor, Ras/Rab), SAPK (p38, Rho/Cdc42/Rac, JNK, p53, TGF-β, TNF-α), and Other (NF-κB, Wnt, JAK/STAT, PI3K/AKT).

There were more single-pathway annotated genes within the Classical (*n* = 15) or Other (*n* = 11) families than SAPK (*n* = 7) in P15 animals. This was in contrast to P30, where more SAPK genes (*n* = 27) were expressed than either Classical (*n* = 19) or Other (*n* = 23) families. Among genes differentially expressed in both P15 and P30, there were less SAPK (*n* = 4) genes differentially than Classical (*n* = 11) or Other (*n* = 7).

**FIGURE 6:**
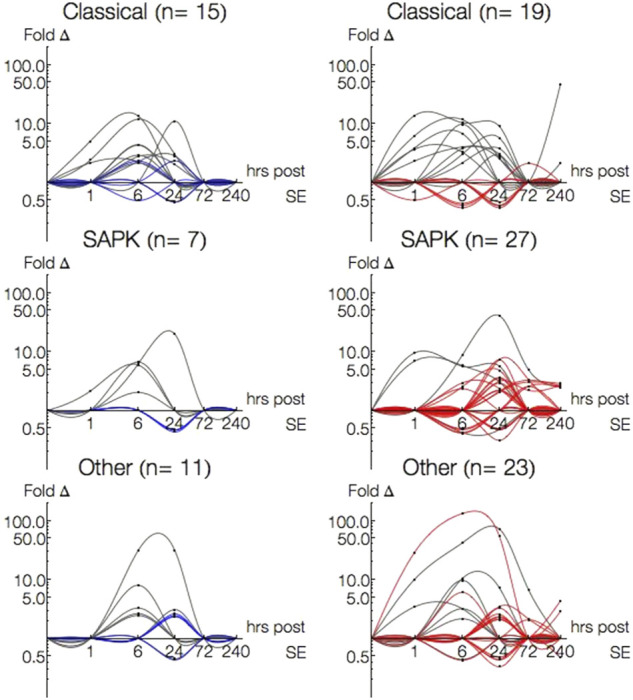
Log-Log plots of differential gene expression (fold change) of single-pathway associated genes amongst Classical, SAPK and Other family groupings. Data points are indicated by black circles. Lines, each representing a single gene, were fitted using second order interpolation for ease of visualization. Genes differentially expressed in both P15 (left column) and P30 (right column) are indicated in gray; Genes differentially expressed only in P15 are blue and only in P30 are red.

Figure 6 plots the single-pathway annotated gene expression profiles of P15 (left column) and P30 (right column) by Classical, SAPK, and Other family groupings. To discriminate between 1 and 6 h, time is presented in log scale. Genes differentially expressed in both age groups are traced in gray, while genes solely in P15 are traced in blue and in P30 traced in red. More genes were differentially expressed at more time points with higher magnitudes in P30 than P15 across all three family groupings. This difference was most striking amongst Other families. In P15 animals, Classical (Cyr62, Bdnf, Rasl11b) and SAPK (Ptgs2) began to be activated 1 h after KA-SE and all genes returned to baseline by 72 h, while Other genes were differentially expressed at 6 and 24 h alone.

Classical, SAPK and Other were all differentially expressed 1–240 h in P30 animals. Most Classical genes were upregulated within 24 h (*n* = 5, 1 h; *n* = 9, 6 h; *n* = 7, 24 h) and most SAPK genes were upregulated 24 h and beyond (*n* = 15, 24 h; *n* = 7, 72 h; *n* = 5, 240 h). Additionally, most Classical genes upregulated within 24 h in P30 were also upregulated in P15, while most SAPK genes upregulated at 24 h and beyond in P30 were not differentially expressed in P15. To investigate what kinds of interactions may be accompanying the overactivation of single-pathway annotated genes in SAPK and Other groupings in P30, we turned our attention to multiple-pathway annotated genes.

### 3.8 Multiple-pathway annotated genes

Forty one percent of both P15 (*n* = 23/56) and P30 (*n* = 48/117) pathway-annotated genes were associated with a variable number (2–7) of multiple pathways. For example, NF-κB1, upregulated in P15 (2.15 fc, 6 h), is annotated with both “TGF-β signaling pathway” and “NF-κB cascade.” Fos, upregulated in both P15 (6.87 fc, 1 h; 16.8 fc, 6 h) and P30 (29.1 fc, 1 h; 24.8 fc, 6 h; 5.82 fc, 24 h), is annotated with four different pathways: “activated by p38 MAPK signaling,” “activated by classical MAPK signaling” (also known as ERK), “TGF-β signaling pathway,” and growth factor signaling (“nerve growth factor pathway (NGF)” and “PDGF signaling pathway,” “PDGFR-α signaling pathway”).

In both age groups, multiple-pathway annotated genes were downregulated only 6 (*n* = 1, P15; *n* = 5, P30) and 24 h (*n* = 2, P15; *n* = 5, P30), and upregulated 1 (*n* = 7, P15; *n* = 11, P30), 6 (*n* = 15, P15; *n* = 25, P30) and 24 (*n* = 7, P15; *n* = 21, P30) hours after KA-SE. Only Ccl2 (5.31 fc, 72 h) and Spp1 (7.37 fc, 72 h; 5.56 fc, 240 h) were differentially expressed after 24 h in P30 (plots not shown). All downregulated genes in P15 and 9 out of 10 downregulated genes in P30 are annotated with only 2 pathways.

**FIGURE 7:**
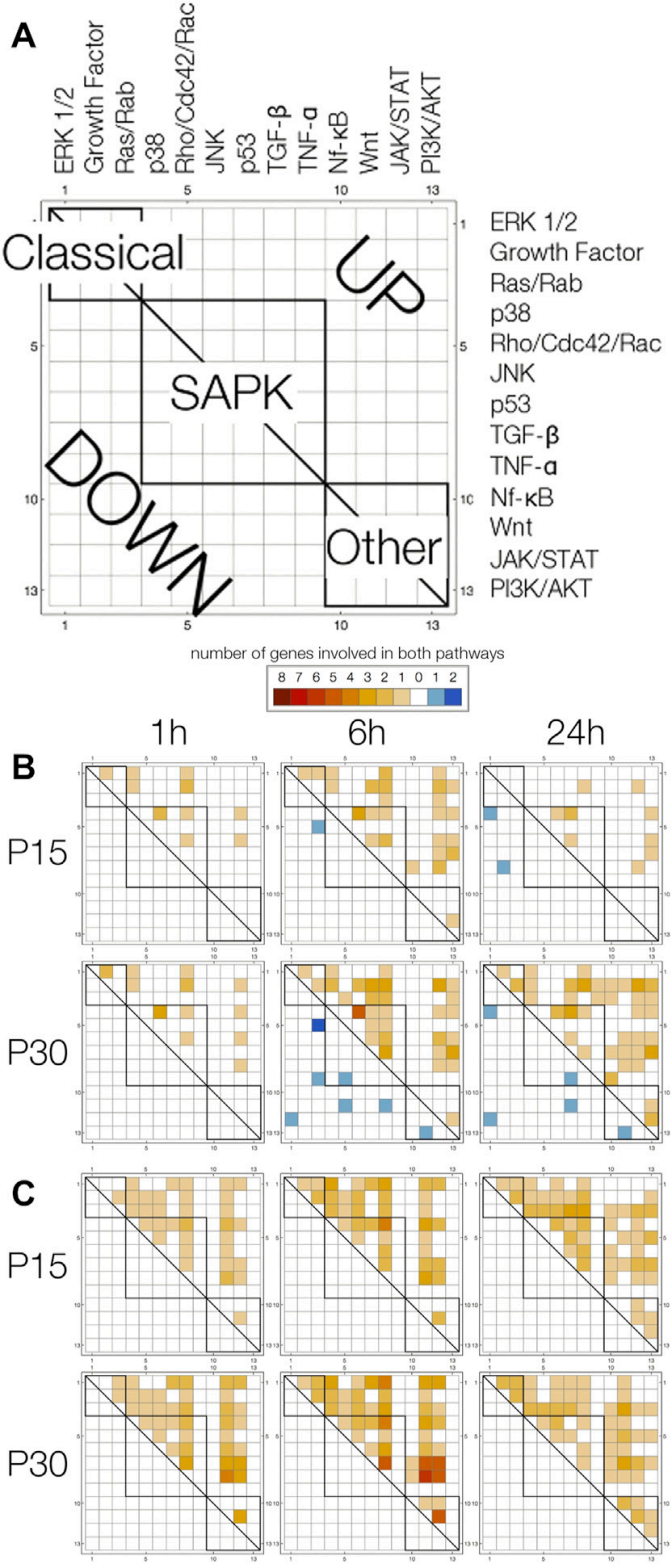
Pathway array plots for multiple-pathway annotated genes plot the number of genes annotated with any two pathways. **(A)** General label for all plots. Pathways are arranged (1 = ERK1/2… 13 = PI3K/AKT) by Classical, SAPK and Other families, and boxes are drawn to indicate pathways are in the same family (intra-family v. inter-family). Upregulated genes are filled in on the upper triangle in a shade of orange, and downregulated genes are filled in on the lower triangle in a shade of blue. **(B)** Pathway array plots for (4 or less)—pathway annotated genes differentially expressed at 1, 6 and 24 h in P15 and P30 animals. **(C)** Pathway array plots for (5 or more)—pathway annotated genes differentially expressed at 1,6 and 24 h in P15 and P30 animals.

To use pathway annotations as a window into possible pathway interactions, we devised pathway array plots (Figure 7A). These display the number of upregulated (upper triangle) and downregulated (lower triangle) genes annotated with any two pathways (1 = ERK1/2… 13 = PI3K/AKT). Since the array plot is two-dimensional, a single gene associated with 2 pathways fills in 1 square, 3 pathways fill in 3 squares, 4 pathways fills in 6 squares, and n pathways fills in n choose 2 squares. Due to the large number of squares filled in for a single gene annotated with 5, 6 or 7 pathways (*n* = 7, P15; *n* = 10, P30) compared to genes annotated with 2 (*n* = 10, P15; *n* = 23, P30), 3 (*n* = 2, P15; *n* = 8, P30) or 4 (*n* = 4, P15; *n* = 7, P30) pathways, we looked at them separately (Figures 7B,C).

Differences between P15 and P30 are clearer amongst genes annotated with 4 or less pathways (Figure 7B). Particularly, in P30 animals, downregulation of genes annotated with multiple pathways at 6 h was followed by increased upregulation at 24 h. At 1 h, P15 and P30 pathway plots are comparable. At 6 h, the major difference between the age groups is that downregulated genes in P30 (Dact2_predicted, Kit, Mtch2_predicted, Plcb4, Ralbp1, RGD1565616_predicted) are annotated with pathways spanning Classical, SAPK and Other families; in contrast, the number of downregulated genes spanning more than 1 pathway is limited to one coordinate for P15. By 24 h, P15 pathway array plot is sparser for upregulated genes compared to P15 6 h. Downregulated genes (Agt, Inha) are still in the Classical and SAPK region. This is in contrast to P30, where downregulated genes (Agt, Faim2, Kit, Plcb1, Prkcc) continue to span all three families. Although upregulated genes in the array plot are sparser within the SAPK box, they are denser for the Other regions. Within the first 24 h, all 5, 6, or 7-pathway annotated genes were upregulated in both age groups. Pathway array plots of P15 and P30 are both dense and comparable: at 1 h, only TNF-α, JAK/STAT and PI3K/AKT have no annotations, and by 24 h, only TNF-α has no annotations (Figure 7C).

### 3.9 Regulation of pathways—Kinases and phosphatases

**FIGURE 8:**
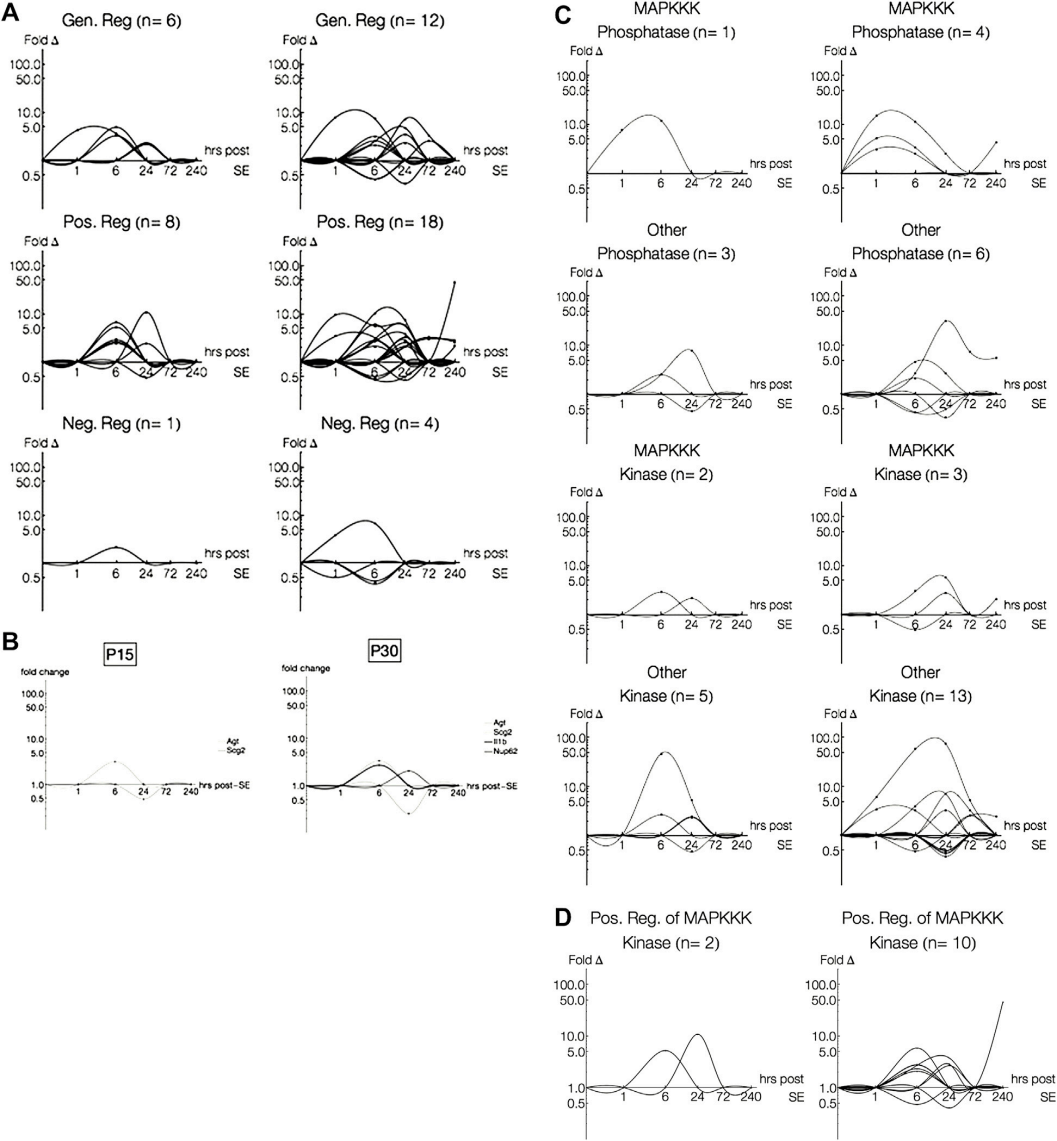
Log-Log plots of differential gene expression (fold change). Lines, each representing a single gene, were fitted using second order interpolation for ease of visualization. **(A)** Log-Log plots of differential gene expression (fold change) of genes regulating pathways. Most genes differentially expressed in P15 (left) and P30 (right) only regulate a single pathway, even amongst multiple-pathway annotated genes. **(B)** Log-Log plots of differential gene expression (fold change) of genes regulating pathways. Only four genes differentially expressed in P15 or P30 were annotated as regulating multiple pathways. **(C)** Log-Log plots of differential gene expression (fold change) of phosphatases and kinases in P15 (left) and P30 (right). **(D)** Log-Log plots of differential gene expression (fold change) of positive regulators of phosphatases and kinases.

Next, we were interested in how the MAPK pathways may be regulated differently between P15 and P30 animals. As mentioned earlier (Section 2.4), pathway annotations will sometimes include regulatory schemes. We isolated genes annotated to regulate the 13 pathways (e.g. “regulation of Wnt receptor signaling pathway,” “regulation of Ras protein signal transduction”), and separated those explicitly annotated as “positive” or “negative” (e.g. “negative regulation of JNK cascade”) regulators. Genes annotated as both positive and negative regulators of the same pathway were not included as “positive” or “negative” regulators and, instead, were grouped as “general” regulatory genes (Figure 8A).

Nearly a third of all pathway-annotated genes in both P15 (*n* = 18/56) and P30 (*n* = 39/119) were regulatory. Most of these regulatory genes only regulated a single pathway (*n* = 16, P15; *n* = 35, P30), even amongst multiple-pathway associated genes. Like the expression patterns for previous groupings, genes annotated as regulating pathways were more numerous and expressed for longer time courses with higher magnitudes in P30 animals. The robust gene induction occurs within 72 h, while a few genes persist at 240 h. This expression pattern is especially evident amongst genes classified as positively regulating a pathway. In P15 animals, positive regulators were upregulated only at 6 h (Cited2, 6.57 fc; Hmox1, 2.43 fc; Jak2, 5.23 fc; Ret, 2.88 fc; Sgk, 2.62 fc) and 24 h (Cd74, 10.85 fc; Tbk1, 2.41 fc), while in P30 animals, positive regulators were upregulated throughout all 10 days of the study. In addition, all genes upregulated in P15 at 6 h were also upregulated in P30, and remained so for longer time courses. For example, Hmox1 is upregulated at 6 (10.3 fc) and 24 h (7.26 fc), and Ret is upregulated at 6 (2.99 fc), 24 (5.68 fc) and 240 h (2.12 fc) after KA-SE.

Rgs4 (2.11 fc, 6 h), which negatively regulates ERK signaling, was the only negative regulator differentially expressed in P15 and the only such negative regulator upregulated in P30 (3.83 fc, 1 h; 6.69 fc, 6 h). Interestingly, this earlier activation of a negative regulator of ERK in P30 (compared to P15) precedes later activation at 240 h of positive regulators of ERK, Ret (2.12 fc) and Cd74 (45.6 fc).

Additionally, we found four genes (*n* = 2, P15; *n* = 4, P30), which regulate multiple pathways (Figure 8B): Agt (“positive regulation of p38” and “positive regulation of classical MAPK”), Scg2 (“negative regulation of JNK cascade” and “positive regulation of TGF-β receptor signaling pathway”), IL-1β (“positive regulation of p38 MAPK cascade” and “positive regulation of JNK cascade”) and Nup62 (“regulation of Ras protein signal transduction,” “positive regulation of epidermal growth factor receptor signaling pathway,” “positive regulation of i-κB kinase/NF-κB cascade”). Of the genes regulating multiple pathways, those differentially expressed in both P15 and P30 were expressed at lower levels than those that were only expressed in P15 or P30.

We were surprised to find that dual specificity protein phosphatases (Dusps), which are key inactivators of MAPK cascades, were not included as negative regulators of any of our pathways. To pursue the question of how phosphatases and kinases may be expressed and regulated differently among all MAPK signaling genes (*n* = 140), we isolated genes involved with phosphatase and kinase activity (Figures 8C,D). Within these genes, we found that 1) some were regulators of kinase and phosphatase activity and 2) some were associated specifically with MAPKKK cascades. All of the MAPKKK associated phosphatases were dual specificity protein phosphatases (Dusp1, P15; Dusp1, Dusp5 and Dusp6, P30).

Although most differentially expressed genes coding for phosphatases and kinases were not associated with MAPKKKs, we see an interesting temporal relationship in both age groups (Figure 8C): Dusps are activated before MAPKKK-related kinases (Ret and Mapk14, P15; Ret, Mapkapk3, Map3k12, P30) and other phosphatases. Similar to what we saw in the array plots and within our pathway regulators, increased Dusp activation earlier in P30 is followed by increased MAPKKK-related kinase activation later (Ret).

The greatest difference between P15 and P30 is across the non-MAPKKK kinases. These kinases are activated earlier and span 1–240 h in P30 but are restricted to 6 and 24 h in P15. However, when we look at the regulators of kinases and phosphatases, we see that the most striking difference between P15 and P30 is across the positive regulators of MAPKKK-annotated kinases (Figure 8D).

### 3.10 Verification

We verified our microarray data in several ways. First, we performed qRT-PCR. Second, we stained for proteins (HSP70 and CD74) coded by two highly expressed genes in P30. Lastly, we performed microarray analysis on tissue collected from hippocampi of human patients with medically intractable MTLE.

#### 3.10.1 Real time reverse transcriptase polymerase chain reaction verifies microarray data for P30 at 24 h

**FIGURE 9:**
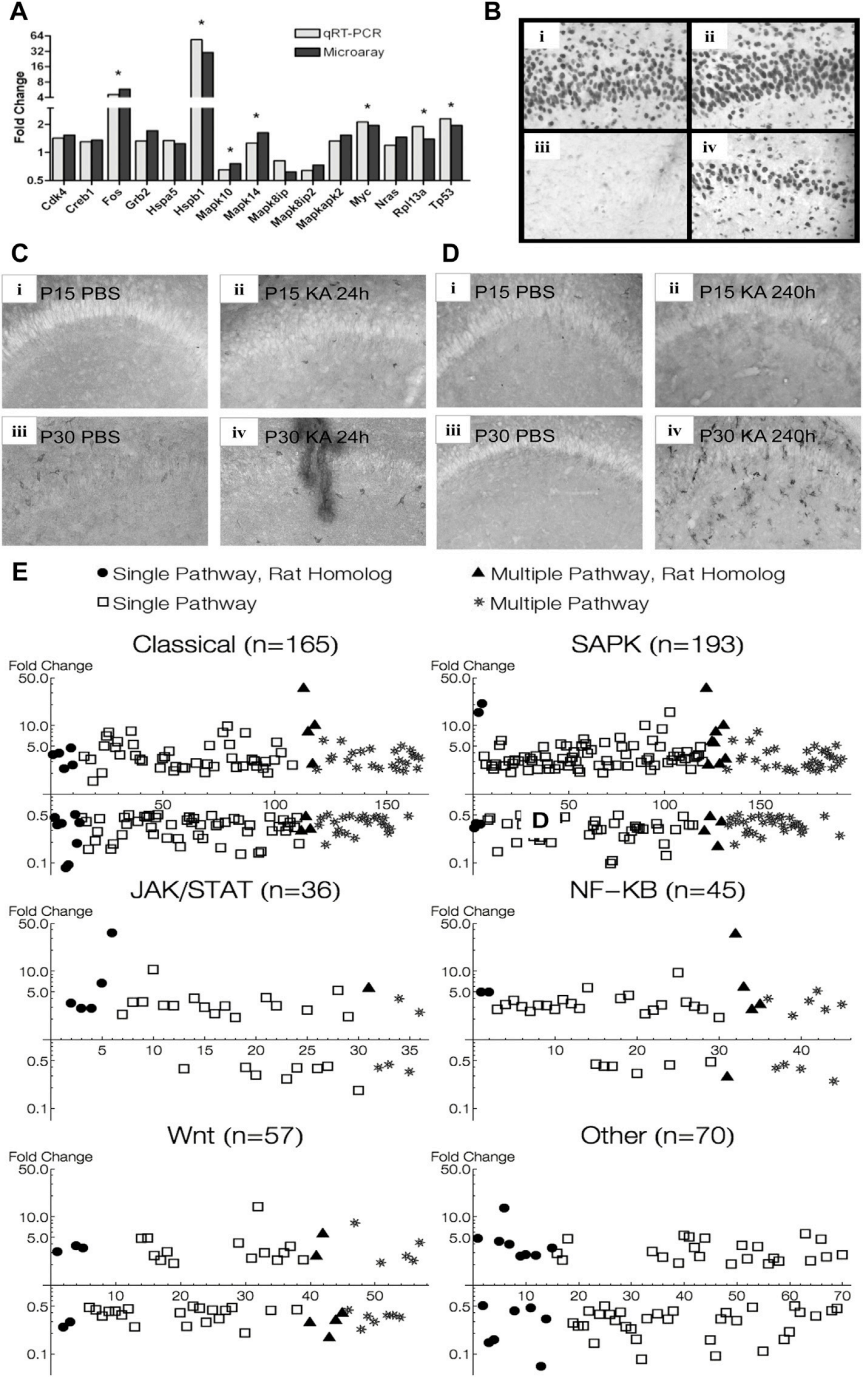
Verification of MAPK pathway genes found in animal experiments. **(A)** qRT-PCR verification of microarray data of some differentially expressed MAPK pathway genes 24 h after KA-SE in P30 animals. **(B)** Hippocampal sections stained for Hsp70. High magnification (×20) view of CA1 subfield 24 h after KA-SE at P15 and P30 [**(i)** P15 PBS; **(ii)** P15 KA; **(iii)** P30 PBS; **(iv)** P30 KA]. Notice robust Hsp 70 immunoreactivity in P15, undetectable in control P30, and induction by KA-SE at 24 h in P30. **(C)** Hippocampal sections stained for CD74 24 h after KA-SE [**(i)** P15 PBS; **(ii)** P15 KA; **(iii)** P30 PBS; **(iv)** P30 KA]. **(D)** Hippocampal sections stained for CD74 240 h after KA-SE [**(i)** P15 PBS; **(ii)** P15 KA; **(iii)** P30 PBS; **(iv)**P30 KA]. CD74 immunoreactive cells are most notable in P30 240 h after KA-SE. **(E)** MAPK pathway genes found in microarray analysis of hippocampus from patients with MTLE superimposed on MAPK pathway genes found in rats after KA-SE. Human genes: open square = single-pathway associated gene; star = multiple-pathway associated gene. Rat genes: filled circle = single-pathway associated gene; filled triangle = multiple-pathway associated gene.

Out of the 84 genes present in the Superarray qRT-PCR array, 61 were also present in the Affymetrix Microarray. Nineteen out of these 61 genes were significantly regulated in P30 microarray data at 24 h (*p* < 0.05, q < 0.05). 15 out of these 19 genes also showed a similar trend in the qRT-PCR data, and 7 of these reached statistical significance, 5 of which were over 2-fold change (Figure 9A).

An additional 16 genes showed statistical significance in our qRT-PCR data, which were not present in our microarray. These included cyclins (Ccna1, Ccna2, Ccnb1, Ccnb2) and cyclin dependent kinase and inhibitors (Cdk2, Cdkn1a, Cdkn2c). Also, we saw MAP2Ks, their activators (Map2k1ip1, Map2k2, Map2k6) and Map3k4. Furthermore, ERK5 showed differential expression at 24 h, suggesting that the fourth prototypical MAPK pathway is also differentially regulated after seizures. Finally, there were Stratifin (which activates MMP1 through p38 and c-fos), Smad4 (which is involved in TGF-β signaling), Rp1p1 and E2f1.

#### 3.10.2 Immunohistochemistry

Hsp70 immunohistochemistry in the rat hippocampus paralleled expression profiles of Hsp 70 in microarray analysis. No difference in Hsp 70 immunoreactive cells are noted between KA and PBS controls in P15 animals, whereas Hsp 70 immunoreactive cells are clearly visible only 24 h after KA-SE in P30 animals (Figure 9B). There appears to be developmental upregulation of Hsp 70 at P15 with subsequent downregulation of Hsp gene expression at P30. Thus, there was no significant changes in Hsp 70 gene expression after KA-SE in P15 at any time point sampled, while Hsp70 (Hspa1a, Hspa1b) was differentially expressed in P30 animals at 1 (28.5 fc), 6 (129.6 fc), and 24 (54.8 fc) hours after KA-SE. It is worth noting that Hsp70 was one of the most upregulated genes, likely reflecting gene induction, at all 3 time points sampled in P30 animals.

We stained for CD74 in P15 and P30 animals at both 24 and 240 h after PBS or KA injection. Immunohistochemistry for CD74 also paralleled changes seen in microarray. 24 h after KA-SE, but not in PBS controls, P15 hippocampi showed staining for CD74. There were some CD74 immunoreactive cells in P30 hippocampi in PBS controls and there appeared more staining in KA-SE animals (Figure 9C). No immunoreactive cells were found 240 h (10 days) after KA-SE and PBS in P15 animals (P25 at the time of sacrifice). In contrast, P30 animals injected with KA showed considerable CD74 staining 240 h after KA-SE (Figure 9D). Although only the hippocampi are pictured in Figure 9, we saw marked increases in CD74 staining throughout all cortical areas 240 h after KA-SE in P30 animals. CD74 is differentially expressed in P15 (10.85 fc) exclusively 24 h after KA-SE. CD74 is slightly upregulated at 24 h (2.84 fc) but it is one of the most highly upregulated genes 240 h after KA-SE in P30 animals (45.6 fc).

#### 3.10.3 Human microarray data on MAPK pathway

Microarray analysis of transcriptional regulation in the hippocampus of human patients diagnosed with temporal lobe epilepsy showed several similar changes in expression of the MAPK associated pathways analyzed in rats (Figure 9E). Several common genes were differentially regulated in both human and rat, with overlap between Classical, SAPK, JAK/STAT, NF-KB, and WNT groupings. The Classical and MAPK groupings showed the most notable changes. Most of the genes differentially regulated (particularly >5-fold change) belonged to either Classical or SAPK groupings. NF-κB, JAK/STAT, and WNT also showed changes, with NF-κB showing the highest proportion of upregulated genes among those differentially expressed. Notably, all pathways were both up and down regulated.

## 4 Discussion

Our first goal was to elucidate the multifaceted immune responses induced by seizures in KA-SE rat models of MTLE and in patients treated surgically for intractable MTLE. We found many common proinflammatory genes upregulated in both rat and human tissue. Some significant differences exist likely because we are comparing temporally discrete gene expressions changes after KA-SE in rats with chronic changes in the MTLE patients who have been exposed to recurrent seizures over many years. It should also be noted that our human control tissues were neocortical tissue while the experimental tissues consist of epileptogenic hippocampal tissue and lateral temporal neocortex. Many of the same genes were upregulated in the temporal neocortex and the hippocampus but to a higher level in epileptogenic hippocampus, lending support to the idea that many of the same inflammatory processes occur in both areas after exposure to chronic seizures. Similarly, higher, and sustained levels of inflammatory genes were expressed in P30 rat hippocampi while lower and transient inflammatory responses were noted in P15 rat hippocampi. Temporal pattern of changes in immune gene regulation provided important information. Acute early changes were activity-regulated transcription factors and immediate-early genes (IEGs) capable of modulating signaling cascade, pathways and protein networks downstream. These transcriptional changes in inflammatory genes in the mature brain (twice more compared to the immature brain) initiate genomic responses that may underlie long-term modification of neuronal physiology. Thus, prolonged seizure activity is translated via gene expression regulation into lasting cellular changes underlying epileptogenic process.

Many immune molecules in the brain have dual functions—acute inflammatory responses and long-term modulation of synaptic activity. The nonspecific inflammatory damage and the more specific modulation of synaptic strengths both have potential to increase cortical excitability. This process seems to be driven primarily by glial cells. The activation of microglia and astrocytes in response to seizure activity is well documented in rats (Shapiro et al., 2008) and gliosis is one of the classic neuropathologic findings in human MTLE. We confirm that glial activation persists over 10 days after status epilepticus in mature rats (Somera-Molina et al., 2007), and demonstrate a similar robust glial activation in both the hippocampi and the lateral temporal neocortex of MTLE patients. We saw persistently high levels of GFAP, Vimentin, and S100B, (astrocyte markers) as well as marked upregulation of both osteopontin and Aif-1, the markers of microglial activation.

IL-1β has been considered the main mediator of innate immune system activation in response to seizures. IL-1β and its receptor are found in neurons, astrocytes, and microglia. Both activated microglia and astrocytes produce IL-1β within hours of seizures, but prolonged production is maintained by astrocytes (Ravizza et al., 2008). In addition to IL-1β, we identified several other cytokines that may propagate the cycle of glial activation. Cytokine upregulation in rats peaked between 6 and 24 h after KA-SE correlating with the peak of glial activation. It is interesting to note that IL-18 expression increased again at the 10-day time point in P30 rats, likely a result of continued microglial activation. IL-1β and IL-18 were also upregulated in the human hippocampus while IL-6 expression was only increased in rats. IL-1β is the only cytokine also upregulated in the lateral temporal neocortex, highlighting its central role in epilepsy. IL-18 is a cytokine of the IL-1β family which is expressed by microglia after KA-induced excitotoxic damage (Jeon et al., 2008). IL-6 is produced by a variety of cells but astrocytes are the dominant source. It has both protective and damaging action including neuroprotection from glutamate toxicity and induction of neurotrophic, inflammatory, and other immune molecules (Van Wagoner and Benveniste, 1999). TNF-α functions in synaptic scaling in addition to promoting inflammation (Stellwagen and Malenka, 2006). It is produced by glia in response to changes in synaptic activity and is necessary for increasing synaptic strength by stimulating cell-surface expression and clustering of AMPA receptors. LITAF is a transcription factor that positively regulates the NF-κB pathway and increases expression of TNF-α. LITAF and TNF-α were upregulated in both rats and human tissue.

The chemokine-mediated recruitment of astrocytes, microglia, monocytes, and other immune cells also plays a central role in the inflammatory response to seizures. We demonstrated extremely high levels of chemokine expression, which generally peaked at 6 h after KA-SE in rats, with the exception of Ccl2, was complete by 24 h. These genes seem to play important roles in the innate as well as the adaptive immunity. The alpha chemokines include Cxcl10, IL-8, and Cxcl2. Cxcl10 was only upregulated in rats while IL-8, and Cxcl2 were only upregulated in our human tissue. Cxcl10 is produced by a variety of cell types, including neurons (Klein et al., 2005), astrocytes, and microglia (Ren et al., 1998). In addition to its functions in the chemotaxis of T-lymphocytes, natural killer cells, and monocytes, Cxcl10 causes astrocyte chemotaxis when upregulated in response to cortical ischemia (Wang et al., 1998). The other two significantly upregulated alpha chemokines in human tissue, IL-8 and Cxcl2, function primarily to stimulate neutrophil chemotaxis and extravasation (Zhang et al., 2001). The beta chemokines, which include Ccl2, Ccl3, and Ccl4, are produced by astrocytes and microglia. They work to increase the migratory response of microglia and monocytes (Peterson et al., 1997). When Ccl2 is overexpressed in the CNS of transgenic mice, it also seems to recruit peripheral monocytes and T lymphocytes (Bennett et al., 2003). To summarize, rats exposed to status epilepticus primarily upregulate the beta chemokines, which function in the chemotaxis of monocytes, microglia, and lymphocytes. However, CXCL10 also shows a modest upregulation. Human hippocampal tissue showed the upregulation of both alpha and beta chemokines, including chemokines with activity on neutrophils and astrocytes. Some of these genes were also upregulated in the lateral temporal neocortex. The broader chemokine profile of the human tissues may be due to continued seizure-induced damage over long periods of time.

Secretoneurin is produced by endoproteolytic processing of Secretogranin II and is found in a variety of neuroendocrine tissues. Its functions include promoting the chemotaxis and extravasation of neutrophils and monocytes (Kahler et al., 2002). In addition, secretoneurin is a chemoattractant protein for endothelial cells, promotes angiogenesis, and stimulates neurite growth in cerebellar granule cells (Fischer-Colbrie et al., 2005). It is also upregulated in the cortical tissue of mice in response to experimentally induced ischemia and seems to have neuroprotective properties (Shyu et al., 2008). Secretogranin II was upregulated in our rat hippocampal tissue with a similar temporal profile as the chemokines.

COX-2 is the rate-limiting enzyme in the production of thromboxane A2 and a variety of prostaglandins. It is one of the key molecules bridging synaptic plasticity and inflammation. A variety of studies have found that COX-2 is expressed at high levels in the normal cortex and hippocampus but also upregulated in response to synaptic activity and seizures (Yang and Chen, 2008). The expression of COX-2 is regulated by NMDA receptor-dependent synaptic activity (Yamagata et al., 1993) and its function is necessary for the induction of long-term potentiation in hippocampal dentate granule cells (Chen et al., 2002). We demonstrate high levels of COX-2 expression in human and rats acutely after seizures. In the human tissue, there was significant upregulation in both hippocampal tissue and the temporal neocortex. The COX-2 produced by cortical tissue in response to seizure activity may induce subsequent inflammatory responses, which in turn may cause increased cortical excitability and further inflammation. Different forms of Phospholipase A2, which also function in promoting inflammation through the eicosanoid pathway, were also upregulated in both the human and rats but their function in the CNS has not been thoroughly investigated.

A wide variety of adhesion molecules were upregulated in both our human and rat tissue. These seem to fall broadly into two categories, those that affect neurite outgrowth and those that have more general immune functions. CD44 binds hyaluronan and is thought to be involved in a variety of inflammatory processes (Pure and Cuff, 2001). It is ubiquitously expressed on leukocytes as well as parenchymal cells and functions in leukocyte recruitment, cell-matrix interactions, cell migration, and induction of inflammatory gene expression. In addition to these roles in immunity, CD44 also functions in axonal pathfinding during development (Lin and Chan, 2003) and high levels of expression have been correlated to mossy fiber sprouting after status epilepticus in mice (Borges et al., 2004). Inhibition of CD44 activity seems to inhibit mossy fiber sprouting in organotypic hippocampal slices exposed to kainite (Bausch, 2006). CD44 was strongly upregulated in the rat hippocampus, peaking at 24 h after KA-SE, a similar time course to mossy fiber sprouting. It was also upregulated in the human TLE hippocampus and temporal lobe neocortex. Celsr proteins are neuron-neuron adhesion molecules that also function in axonal pathfinding during development (Tissir et al., 2005). Gene silencing experiments demonstrate that Celsr3 inhibits neurite outgrowth, while Celsr2 stimulates it (Shima et al., 2007). In adult rats, Celsr3 is one of the few molecules to be significantly downregulated in the period up to 24 h after KA-SE, suggesting the therapeutic potential of Celsr3 in preventing the formation of aberrant synaptic connections. Contactin 2, also called TAG-1, is an immunoglobulin superfamily member expressed by neurons, which promotes neurite outgrowth (Furley et al., 1990). It is anchored onto the neuronal membrane, and also secreted into the extracellular space where it may function as a substrate adhesion molecule. Expression of Contactin 2 was only significantly increased in the human MTLE patients. The tetraspanins, a group of cell-surface adhesion molecules, have a multitude of roles in cell motility, integrin-dependent cell adhesion, cell proliferation, apoptosis, tumor metastasis, and in organizing cell surface signal transducing complexes, or microdomains (Hemler, 2005). Leukocytes contain up to 20 different tetraspanins, which interact with a variety of immune molecules, including CD4, CD8, Fc receptors, MHC I, and MHC II molecules (Tarrant et al., 2003). The tetraspanins upregulated in rat, CD9, CD37, CD53, and CD63 are found in B-cells, T-cells, monocytes/macrophages, and granulocytes. These molecules showed an interesting pattern of expression. CD37 and CD53 are upregulated primarily at the late 72 h and 240 h time points while the other tetraspanins peaked at 24 h, suggesting a multiphasic immune response. Our human MTLE data also demonstrates upregulation of CD9 and CD53 in addition to Tetraspanins 6, 8, and 15. This group of molecules may also contribute the tissue-leukocyte interactions that lead to immune system activation.

Other adhesion molecules found to be expressed in rats and human function in leukocyte extravasation. They tend to be upregulated in response to inflammatory cytokines and are found either on leukocyte membranes or endothelial surfaces. These include L-selectin, ICAM-1, and the integrins. Recent studies demonstrate that molecules such as ICAM-1, VCAM-1, and selectins are upregulated in response to seizure activity and that disruption of their interactions with leukocyte receptors such as integrins prevents BBB breakdown and leukocyte extravasation (Fabene et al., 2008). Further highlighting the therapeutic potential of disrupting the inflammatory response to seizures, disruption of leukocyte extravasation led to decreased frequency of seizures after pilocarpine-induced SE. A number of receptors that function in the adaptive immune system were also upregulated in our experiments. This may suggest a breakdown in the BBB and infiltration of peripheral leukocytes. Indeed, chronic expression of IL-1β in the mouse brain leads to blood brain barrier breakdown, expression of CCL-2, and a dramatic infiltration of neutrophils, T-cells, macrophages, and dendritic cells (Shaftel et al., 2007; Xu et al., 2018). Chronic IL-1β expression leads to neutrophil infiltration of the hippocampus persisting up to 1 year due to activation of the CXCR2 receptor. Histopathologic examination of surgical specimens from children with intractable epilepsy also shows BBB disruption due to an angiopathy of capillaries and arterioles (Hildebrandt et al., 2008).

Another important goal of our research was to delineate some of the differences in status epilepticus-induced gene expression changes between mature and immature rats. P15 rats have increased seizure susceptibility in response to electrical stimulation (Moshe and Albala, 1983). Similarly, systemic injections of KA causes more severe seizures and a higher rate of death in immature rats but they are paradoxically less susceptible to the development of convulsions later in life (Okada et al., 1984). Furthermore, immature rats are much more resistant to neuronal death, mossy fiber sprouting, and the synaptic reorganization that alters the electrophysiological properties of the hippocampus (Haas et al., 2001). Our data showed a peak in expression of both astrocyte and microglial markers at 24 h after KA-SE in mature as well as immature rats. However, the upregulation of these markers in immature rats was largely terminated by 72 h, whereas the mature rats have higher peak expression levels and upregulation is significantly prolonged, lasting through the 240 h time point. We propose that temporal expression difference may be responsible for the increased damage and epileptogenesis seen in mature animals. We also observed a dramatic discrepancy between cytokine, chemokine, complement, and protease expression between these groups. The increased seizure susceptibility seen in immature animals may be due to active synaptogenesis and synaptic plasticity in young nervous system, but seizures without the prolonged inflammatory responses do not seem to produce the long-term changes seen in mature animals.

MAPK signaling comprised the largest functional grouping for genes differentially expressed in P30 and for genes differentially expressed in P15 (Figure 5A). These genes were also associated with other processes (e.g., inflammation, synaptic plasticity) previously observed to change during the latent period, suggesting that differential regulation of MAPK signaling may underlie such functional alterations in older animals (Figure 5B). Using online databases, we found that MAPK genes differentially expressed in response to seizures in P15 or P30 fell into numerous canonical signaling schemes, which we reduced to 13 component pathways (Growth Factor, Ras/Rab, ERK, p38, JNK, Rho/Rac/Cdc42, TGF-β, TNF-α, NF-κB, Wnt, JAK/STAT, PI3K/AKT) based on the criterion that each pathway contained a unique gene. After this simplification, most genes expressed in P30 or P15 were associated with only a single pathway. However, most genes within any given pathway were still associated with multiple pathways, suggesting that genes differentially expressed in response to seizures (P15) and during the latent period (P30) can participate in multiple canonical signaling cascades. This may allow such genes to act as central regulatory hubs, whose dysregulation can have propagating effects across the signal transduction network. Compared to P15, P30 animals also showed marked increases in genes participating in positive regulation at multiple levels of signal transduction: at the level of pathways (Figures 8A,B), at the level of transcription (Figure 6) and at the level of MAPKKK cascades (Figure 8D). Additionally, increases in downregulation at P30 precede increases in upregulation of multiple-pathway annotated genes (Figure 7B), and increases in negative regulation of ERK signaling precede increases in positive regulation of ERK signaling (Figures 8A,B). This is consistent with the idea that transcriptional feedback into MAPK signaling during the latent period produces a positive feed-forward loop, as we see drastic increases in positive regulation coupled with negative regulatory elements precipitating positive regulation.

Another striking difference between the age groups was unmasked by splitting up our pathway related genes based on MAPK family groupings (i.e., SAPK and Classical). We found that most of the genes in both P30 and P15 animals that belonged exclusively to one of the Classical pathways (ERK, Ras/Rab, Growth Factor) were commonly expressed across age groups and expressed within the first 24 h (Figure 6). At around 24 h, genes belonging exclusively to one of the SAPK pathways (p38, JNK, Rho/Rac/Cdc42, TGF-β, TNF-α), on the other hand, act differently between the age groups. Particularly, we saw genes exclusively expressed in P30 animals were upregulated and remained upregulated for the entire sampled period of 10 days. This, coupled with the late and persistent activation of genes which serve as glial markers and glial activators (S1004a, Ccl2, Spp1, Aif-1), may suggest a network disturbance spanning multiple signaling schemes across multiple cell types, which together propagate a persistent over-activation of transcription in epileptic P30 animals.

Our data support that chronic epileptic state and spontaneous recurrent seizures after KA-SE emerge from a systemic disruption of signal transduction networks across multiple canonical pathways. At the level of transcription, our data suggests this disturbance is propagated by over activation in the direction of feed-forward loops onto MAPK signaling pathways. Studies looking at phosphorylation of p38, JNK and ERK signaling have described different time courses of expression from each other as well as across cell types and hippocampal regions (Gass, 1999; Mielke et al., 1999; Jeon et al., 2000; Berkeley et al., 2002; Brisman et al., 2002; Ferrer et al., 2002; Choi et al., 2003; Kim S. H. et al., 2004; Kim S. W. et al., 2004; Jiang et al., 2005; Houser et al., 2008). However, the results of these experiments have been inconsistent across different models of epilepsy, and even in the same model. Though attributed to methodological differences, this may suggest that seizure-induced directional changes in the regulation of MAPK pathways are not consistently predictable. It is plausible that widespread dysregulation across dynamically modular networks with emergent nonlinear properties may rely more on changes in network dynamics, and less on specific genes or species. We propose that chronic spontaneous recurrent seizures after KA-SE result from a systemic disruption of signal transduction networks across multiple pathways affecting multiple cell types.

MAPK pathways are composed of several different families with different signaling motifs and crosstalk between pathways which can activate or deactivate each other in a context dependent manner (Shen et al., 2003; Aksamitiene et al., 2012; Fey et al., 2012). All of our 13 pathways have previously been linked to various models of epilepsy, and the potential crosstalk between them has been discussed elsewhere (Gautam et al., 2021). Though the role of interaction and crosstalk between pathways still are not well understood, studies suggest that under non-pathological conditions one way of gaining signal specificity and maintaining robustness in signaling transduction systems is through molecular species acting to mutually inhibit one another (Natarajan et al., 2006; McClean et al., 2007), and that most potential interactions between molecules are not realized, limiting cellular responses to discrete subsets of ligands and pathways to enhance specific cellular functions (Hsueh et al., 2009). Our data suggests that in P30 animals, the crosstalk between pathways as well as the pathways themselves are overactivated. Particularly, in comparison to P15 animals, P30 animals have sustained overactivation and further expression of genes specific to P30 across all pathway families (Figures 6–8). We propose sustained overactivation of multiple crosstalking pathways, especially those in our SAPK grouping, may be recruiting instead of inhibiting crosstalking species. This “leakage,” instead of maintaining signal specificity, results in a diverse array of cellular responses, culminating in epileptogenesis.

Single-cell transcriptomics have identified cell-specific changes related to plasticity and inflammation, particularly in interneuron and glial populations (Pfisterer et al., 2020; Kumar et al., 2022). Potential central regulators explaining glial genes expressed during epileptogenesis are directly upstream and regulate several MAPK pathways (Kalozoumi et al., 2018). Interestingly, reports of MAPK related changes in the extracellular matrix (Han et al., 2019) may also suggest that signaling transduction networks perturbed during epileptogenesis operate across cells, especially in neuron-glial interactions. For example, microglia in the dentate gyrus, shortly after SE, have increased IGF-1 expression, mediated by CREB (Choi et al., 2008) and likely MAPK signaling (Labandeira-Garcia et al., 2017; Wu et al., 2018). Two days post SE, reactive microglia near the subgranular zone (SGZ) drop IGF-1 to drive cellular proliferation via ERK in the SGZ (Choi et al., 2008).

There are limitations when comparing gene expression profiles in an acute rat model to human epileptic brains. While we emphasized the commonality between rat and human data, showing that the same inflammatory genes are persistently upregulated in P30 hippocampi and human epileptic tissue, distinct classes of genes uniquely expressed in rats or humans are noteworthy. Heat shock proteins were uniquely upregulated in rats during the first 24 h after KA-SE. Conversely, adaptive immune system activation is noted only in human tissue. These differences reflect acute reactive changes in gene expression in the rat KA-SE model compared to the chronic epileptic state in humans. The body responds to recurrent seizures over time: breakdown of blood brain barrier occurs and immune cells from periphery infiltrate into the brain in chronic epilepsy (Xu et al., 2018). Given this limitation of comparing an acute epileptogenic insult model (KA-SE) to chronic epilepsy, it is remarkable to see many inflammation-related genes and the same MAPK pathway genes altered in parallel in both rats and humans. Our findings thus provide clinical relevance and credence to our preclinical time-course gene expression profiling.

In summary, we demonstrated significant changes in hippocampal gene expression in MAPK signaling pathways after prolonged seizures and identified potential role of dysregulated SAPK and p38 MAPK in pathogenesis unique to P30. We also demonstrated significantly higher levels of inflammatory gene upregulation in the epileptogenic hippocampus compared to the lateral temporal neocortex of patients with MTLE. Age- and time-dependent differential regulation of inflammatory genes after KA-SE further demonstrated that chronic, persistent and active inflammation occur only in epileptic P30 animals while transient inflammation is observed in P15 animals that show no cell death and no spontaneous recurrent seizures. This observation suggests a critical role of chronic active neuroinflammation in epileptogenesis. Under normal physiologic conditions, the immune system likely subserves many of the functions necessary for proper synaptic scaling and so must be responsive to synaptic activity. When appropriately activated and deactivated, inflammation benefits the host defense response by removing damaged cells and promoting repair and recovery. However, prolonged seizures appear to inappropriately activate the immune system leading to sustained, out of control inflammation. This uncontrolled inflammation can exacerbate neuronal injury and cause immediate damage to the brain and long-term changes in brain structure by stimulating neurite outgrowth and the formation of aberrant synapses, thus promoting epileptogenic process.

## Data Availability

The datasets generated for this study are available upon request to the corresponding author. The datasets are also available at the Microarray Consortium (https://www.ncbi.nlm.nih.gov/), accession numbers GSE1834 and GSE1831, and at TGEN (Phoenix, AZ).
